# Automated CAD system for early detection and classification of pancreatic cancer using deep learning model

**DOI:** 10.1371/journal.pone.0307900

**Published:** 2025-01-03

**Authors:** Abubakar Nadeem, Rahan Ashraf, Toqeer Mahmood, Sajida Parveen

**Affiliations:** Department of Computer Science, National Textile University, Faisalabad, Pakistan; University of Manitoba, CANADA

## Abstract

Accurate diagnosis of pancreatic cancer using CT scan images is critical for early detection and treatment, potentially saving numerous lives globally. Manual identification of pancreatic tumors by radiologists is challenging and time-consuming due to the complex nature of CT scan images and variations in tumor shape, size, and location of the pancreatic tumor also make it challenging to detect and classify different types of tumors. Thus, to address this challenge we proposed a four-stage framework of computer-aided diagnosis systems. In the preprocessing stage, the input image resizes into 227 × 227 dimensions then converts the RGB image into a grayscale image, and enhances the image by removing noise without blurring edges by applying anisotropic diffusion filtering. In the segmentation stage, the preprocessed grayscale image a binary image is created based on a threshold, highlighting the edges by Sobel filtering, and watershed segmentation to segment the tumor region and we also implement the U-Net method for segmentation. Then refine the geometric structure of the image using morphological operation and extracting the texture features from the image using a gray-level co-occurrence matrix computed by analyzing the spatial relationship of pixel intensities in the refined image, counting the occurrences of pixel pairs with specific intensity values and spatial relationships. The detection stage analyzes the tumor region’s extracted features characteristics by labeling the connected components and selecting the region with the highest density to locate the tumor area, achieving a good accuracy of 99.64%. In the classification stage, the system classifies the detected tumor into the normal, pancreatic tumor, then into benign, pre-malignant, or malignant using a proposed reduced 11-layer AlexNet model. The classification stage attained an accuracy level of 98.72%, an AUC of 0.9979, and an overall system average processing time of 1.51 seconds, demonstrating the capability of the system to effectively and efficiently identify and classify pancreatic cancers.

## 1. Introduction

One of the most fatal cancers, pancreatic cancer has a dangerous onset, complex nature, and poor prognosis. Due to bad prognosis, reported fatalities of 466000 as cases of 496000 [1]. In 2020, it ranks seventh in terms of death rate from cancer in both genders, including cases of mortality in males (246,840) and females (219,163) [2]. The two most common forms of pancreatic cancer are adenocarcinoma, which accounts for about 85% of cases, and pancreatic endocrine tumors, which account for less than 5% of cases [3–5]. Due to poor diagnosis methods, pancreatic cancer is frequently detected when a treatment is no longer curative at an advanced stage, which causes increased death rates. Therefore, to enhance diagnosis and treatment outcomes, automated systems with early cancer detection capabilities are essential. In the medical field, many different algorithms have been used. To diagnose and treat patients effectively, data must be clear and dependable. There is ample room for the advancement of sophisticated computer diagnostic systems [6]. Pancreatic tumors are one of the leading causes of death worldwide from cancer. This is a result of a lack of the most efficient tools for early detection. These days, pancreatic tumors are analyzed and presented using a lot of data from the automated detection of pancreatic cancers using computed tomography. Because only low-level features can be extracted using conventional methods [7]. Abdominal CT misses about 40% of tumors in the pancreas that are smaller than 2 cm. Thus, this contributes significantly to the failure of pancreatic cancer early detection [8]. Improving PDAC early detection would have a major effect on patients’ prognosis because tumors that were discovered by accident when they were still within the pancreas and less than 2 cm have been known to survive for more than 60% of cases [9].

Automated computer-aided diagnosis systems can help with a faster workflow, shorter reading times, fewer doses and contrast agents, earlier disease detection, better detection accuracy, and more precise diagnostics [10]. In the future, radiologist’s issue more AI-based tools as AI is progressively incorporated into radiography. As AI tools continue to grow at their current rate, it is anticipated that many medical imaging applications will move from the bench to the bedside in increasingly accurate, affordable, and accessible versions [11]. The last decade has seen an exponential increase in interest due to deep learning’s great performance potential in radiology on a variety of computer vision applications, such as segmentation, detection, monitoring, prediction, and classification [12]. CAD has become a most effective tool in the medical area. Through the analysis of vast imaging data in the medical field artificial intelligence (AI), techniques can detect small changes in the pancreas that might go unnoticed by human observer’s eyes. AI-based computer-aided detection (CAD) is a system tool with great potential that helps improve pancreatic tumor detection and response assessment. The development of CAD has accelerated due to deep learning techniques over the past few decades in a variety of fields, and it is anticipated that it will continue to be a catalyst for technological advancement for some time to come [13]. In the past few decades, the use of artificial intelligence has become a prominent area of academic study for medical image interpretation, with an astounding growth in the number of AI studies conducted. Research labs have created thousands of AI radiology solutions, some of which are currently performing on par with or even better than physicians [14, 15]. Still, early diagnosis can be challenging and mostly relies on imaging techniques [16]. CNN is an excellent DL architecture for image-related tasks, and there is no denying that CNN models have contributed significantly to the recent surge in interest in deep learning. CNN’s ability to determine features without human intervention is a major advancement above previous models of this kind. In addition to conducting parameter sharing, CNN is a computationally efficient model that makes use of special convolution and pooling methods. Now that CNN models are available for usage on all platforms, a larger audience finds them to be more appealing [17].

Artificial intelligence has the potential to aid radiologists in the early detection of prostate cancer by leveraging vast amounts of imaging data. Deep learning models are a class of artificial intelligence (AI) algorithms that includes convolutional neural networks (CNNs). They are particularly well-suited for image analysis because they have demonstrated exceptional accuracy in the image-based diagnosis of numerous cancer types [18–20]. The severity of pancreatic cancer makes it imperative to develop CAD systems capable of distinguishing between cancerous and non-cancerous tissue. Therefore, developing an advanced pancreatic cancer discriminating mechanism is crucial. By probing the local spatial correlations in an image, a convolutional neural network (CNN) can extract features from the image. CNN models have demonstrated efficacy in handling a broad range of challenges associated with image classification [21]. Thus, a system that uses the limited dataset of CT scans to automatically identify and classify pancreatic cancers at an early stage with excellent performance must be developed.

The major contributions of this proposed work are:

- We introduced an entirely automated, cutting-edge CAD system architecture for the early identification and categorization of pancreatic cancers.
- Fine-tuning the hyper-parameters of the algorithm to optimize the performance for the specific task of tumor segmentation, detection, and classification.
- To improve the multi-classification of the multimodal medical images of pancreatic cancer by using optimum model training of the reduced 11-layer framework of the AlexNet-CNN.

## 2. Related work

Radiological imaging is vitally important in the detection and management of pancreatic ductal adenocarcinoma (PDAC) yet, it is limited in such ways as being unable to distinguish an absence of cancer after neoadjuvant therapy from a mere tumor response. Computer-aided detection (CAD) based on artificial intelligence (AI) could lessen the burden of detecting cancer, but its uptake is still slow. Specifically, this review discusses current radiological computer-aided detection systems for pancreatic cancer and solicits pressing issues that prevent the growth of clinical practice associated with computer-aided detection systems. AI has described the chance of early detection, monitoring of response to treatment, and assessment of respectability. However, these applications come with challenges, such as dataset quality and result transparency, through which human-centered AI design is compulsory. It is, therefore, recommended that future research focus on these challenges in a bid to advance pancreatic cancer diagnosis and treatment [22]. Unlike radiologists’ interpretation, this study was done to assess the diagnostic performance of the contrast-enhanced computed tomography images that utilized a deep learning method; convolutional neural networks used to differentiate pancreatic cancer. A total of 690 cases, 320 controls, and 370 pancreatic cancer patients were fed for testing and training or validation datasets. The CNN was trained to determine whether an area in the image is malignant or not. CNN exhibited excellent specificity, accuracy, and sensitivity, performing much better than radiologists for both sensitivities. Namely, CNN for tumors < 2 cm was 92.1%. Therefore, this study is indicated for CNN to be used for this purpose because it provided good results in the differentiation of pancreatic cancer in CT scans from other benign masses and other diagnoses, as it could apply to patients with different racial and cultural backgrounds [23]. Present a novel framework using Deep Convolutional Neural Networks (DCNNs) for achieving the localization of pancreatic tumors in contrast-enhanced CT imaging. The proposed framework enhances context usage, a critical trait for precise tumor diagnosis, which conventional approaches and classical CNN models often find difficult to utilize. The system has three main building blocks: a Dependencies Computation (DC) Module, Self-adaptive Feature Fusion, and Augmented Feature Pyramid networks. The Augmented Feature Pyramid networks assist the network in detecting tumors through extraction and propagation of low-level localization information. Self-adaptive Feature Fusion records richer context information at different scales, following predefined areas of interest. The DC Module helps further in increasing the detection accuracy by recording interaction information between tumor proposals and surrounding tissues. All other experimental results of the proposed scheme show more competitive performance than previously proposed state-of-the-art approaches, having an AUC of 0.9455. This highly illustrates that the scheme developed might find the pancreatic cancer tumor in a patient more precisely and effectively [24].

Develop a convolutional neural network (CNN) based deep learning detection system that can automatically discern between normal tissue and pancreatic cancer in CT scans. A dataset including 3494 CT images of patients with pancreatic cancer and 3751 CT images of patients with normal pancreas was used to build a CNN model. Ten-fold cross-validation was used to assess the method for binary and ternary classifications across three datasets that were created based on image phases. With no discernible sensitivity variations throughout the phases, the binary classifier’s overall diagnosis accuracy was 95.47%, 95.76%, and 95.15% on the plain scan, arterial phase, and venous phase, respectively. When it came to plain scan diagnosis, CNN surpassed trainees and performed on par with board-certified gastroenterologists. The overall diagnosis accuracy for the ternary classifier was 82.06%, 79.06%, and 78.80% for the venous phase, arterial phase, and plain phase, respectively. The study found that the created CNN classifier was appropriate for pancreatic cancer screening, indicating the potential for enhancing diagnostic precision in clinical settings [25]. I design an artificial intelligent (AI) model that uses dynamic contrast-enhanced CT images to diagnose pancreatic cancers. The training of the model is based on the dataset, which involves 143,945 records of CT scans covering 319 patients. It is comprised of four stages: image screening, localization of the pancreas, segmentation of the pancreas, and identification of the pancreatic tumor. When tested independently on its own data, the team found the overall sensitivity of the AI system to be 88.5% and an area under the curve at 0.871, according to the report. Overall accuracy was at 82.7%, with very high specificity: the specifics were 100% for intraductal papillary mucinous neoplasm and 87.6% for pancreatic ductal adenocarcinoma. The test time per patient was much faster at an average of 18.6 seconds compared with manual reviewing. The system also generated saliency maps, which highlighted important locations and made diagnoses clearer and hence more easily interpretable. The study concluded that the rapid and accurate preoperatively model diagnosis could facilitate surgical therapy for pancreatic tumors [26]. A multi-scale segmentation-for-classification technique be modified to automatically detect pancreatic ductal adenocarcinoma (PDAC) in CT images of the abdomen. This technique would assist radiologists in the locations of tumors by automatic classification of volumes following tumor segmentations. A coarse-to-fine flowchart is combined with multi-scale inputs which are treated for tumors in different sizes. Post-processing of the modules uses an additional step that can avoid false alarms and filter outliers. The study has the largest known set of PDAC tumors—136 cases, and 303 are normal cases, consisting of a total of 439 CT images. The following framework seems to have a possible clinical impact on the identified sensitivity of 94.1% and specificity of 98.5% [27]. Deep neural network training is made easier using a residual learning framework. The approach allows the training of networks much deeper than prior models by reformulating layers as learning residual functions respecting the layer inputs. These residual networks can be optimized more easily and with better depth and accuracy, according to empirical data. An analysis of the ImageNet dataset reveals that residual networks with up to 152 layers—eight times deeper than VGG nets—achieve an error rate of 3.57% on the ImageNet test set, good enough to win the 2015 ILSVRC classification job. Because of the deep representations that the deep residual nets generate, the study also demonstrates notable gains in visual identification tasks, including a 28% relative improvement in the COCO object detection dataset [28].

The specific feature leads to reusing building blocks, in the aggregation of modifications and their topologies, towards a modernized, modularized network architecture that categorizes images. This thereby leads to creating a homogeneous and multi-branch architecture without excessive hyper-parameters. In this sense, cardinality thus focuses on enunciating the aspect of defining the size of the set of transformations as an important element besides depth and width as put forth by classic ResNet explanations above. All experiments are done on ImageNet-1K, demonstrating that even under complexity constraints, higher cardinality increases accuracy. The proposed model, called ResNeXt, took second place at the ILSVRC 2016 classification challenge. Models and codes are online. Further experiments on the COCO and ImageNet-5K sets demonstrate that ResNeXt performs better than ResNet [29]. Architecture such as numerous branches to improve illustration gaining knowledge of convolutional neural networks (CNNs). To make use of function-map attention and multi-course representation, this law applies channel-wise interest across many branches. Through pass-function interactions, the counseled Split-Attention module gives a sincere and modular computation block that could take the area of the well-liked residual block, resulting in a wider variety of representations. The performance of current designs consisting of RegNet-Y and FBNetV2 can be enhanced by integrating the Split-Attention module. The researchers also provide ResNeSt, a novel version of the ResNet model that outperforms EfficientNet in phrases of the accuracy/latency exchange-off whilst the Split-Attention module is used in the location of residual blocks [30]. Computed a modified convolutional neural network method to increase the effectiveness of clinical images. They modified the convolutional neural network-based AlexNet method so that it could operate in a 512-by-512 input space. The filter sizes of convolutional layers and maximum pooling were both reduced. This modified CNN was used to test and create numerous other strategies. To classify pancreatic absence/presence CT images, better Convolutional Neural Network (CNN) estimates were created. Also correlated was the total accuracy determined on test images that weren’t used to train the Resnet [31]. Instruction can make up for this deficiency on a publicly available data set. For this purpose, advanced (6084) CTs from individuals with PDAC (338) were used as input in combination with a sixteen-layer (VGG16 CNN) model with R-CNN in a more complex version of the two methods for diagnosing PDAC. A combination of VGG16 and R-CNN demonstrated a very high prediction accuracy of around 96%. It takes 0.2 s for R-CNN to process one CT image. it is incomparable and rather lightning-fast by an expert in visual clinical imaging [32]. In its turn, the data led to the further analysis of 82 CT scans of the abdomen by a DL-based network performing the role of a model of a Gaussian mixture with its content from a radiomic dataset with 19000 features. Another advancement related to lump recognition was its proposal of an input into the recognition algorithm: that is to specify the region in which detection of a lump’s growth takes place or the interest region. Each layer of the CNN collects information from that locality, to come up with a model to determine the features of the lump like size, shape, and weight. The findings were useful to the patients by showing how widespread the pancreatic head tumor was after the diagnosis and treatment. However, only those tumors located in the head area, which is the only region caring for the condition, can be examined in this scope of the investigation [33]. For instance, screening utility determined in PCs proved to be of high accuracy in a meta-analysis of the published data, based on AI-endoscopic ultrasound (EUS). Thus, data for conduction of the analysis with the use of artificial intelligence (AI) were retrieved based on 10 trials, combining data from 1,871 patients using convolutional neural networks, artificial neural networks, and support vector machines. The ANN model proved to have the greatest sensitivity in small PC identification, with a range of 93% for Endoscopic Ultrasound (EUS) and 53% for Computed Tomography (CT) to 67% for Magnetic Resonance Imaging (MRI), respectively. These ten studies might be overrated because the internal validity was tested for data generalization. The validation sources should, therefore, include several groups drawn from its entire demographic if these were to apply to patients belonging to such populations [34].

Automatic segmentation methods for pancreatic and pancreatic tumors over the past decade. It focuses on the shift from traditional unsupervised techniques, such as clustering and thresholding, which showed limited success, to advanced deep learning techniques, especially Convolutional Neural Networks (CNNs), which have significantly improved segmentation accuracy, and critical comparisons of different techniques Some advantages include increased efficiency in medical image analysis, but challenges remain, such as the need for large data sets information and computer resources are used Future work should address these limitations to further refine segmentation methods [35]. "AX-Unet," a deep learning framework, was developed to detect pancreatic cancer. Given the aggressive nature of pancreatic cancer, the importance of early detection has been emphasized. AX-UNet combines the U-Net and Attention methods to focus relevant symptoms on the CT scan. It also uses fewer loss functions and optimizers to achieve better segmentation accuracy and precision. It was trained on annotated pancreatic CT data sets with data enhancement. ROC curves and AUC measurements prove the accuracy and efficiency of AX-Unet over current methods. Implications for clinical practice mean improved diagnostic capabilities to the benefit of radiologists. Subsequent research attempts to integrate AX-Unet with other imaging techniques and complex datasets [36]. U-Net and its modifications for pancreatic CT segmentation show various improvements in techniques, focusing on convolution block analysis and attention mechanism integration The results show significant improvements in segmentation accuracy, using deformable diffractions with dice coefficients up to 0.8725 is used. However, it increases the computational complexity and training time of these methods. This systematic review highlights the balance between model accuracy and statistical efficiency, highlighting the challenge of adapting these methods to clinical practice [37]. TSADL-PCSC method combining W-Net segmentation method, GhostNet feature extraction, DESN classification and TSA-based hyperparameter tuning, to improve pancreatic cancer screening results showed improved classification performance on CT scan datasets and included. However, limitations include reliance on extensive training data and potential overfitting due to complex sampling design. This approach has shown remarkable improvement in the automatic detection of pancreatic cancer, but further validation is needed in different contexts to ensure its generalizability [38]. PIS-Unet model that combines the Inception and SE modules with the Pyramid Pooling Module (PPM) to enhance the segmentation of intestinal tumors. The method involves multi-scale feature extraction and adaptive channel weighting, which improves segmentation accuracy. The results of the ablation tests show that PIS-Unet obtained a Dice Similarity Coefficient (DSC) of 87.90% for MCN and 85.49% for SCN, which outperform other methods Improved Benefit segmentation performance due to ROI imaging have reduced the number of accounting jobs and more. Limitations include reliance on recorded data and potential computational complexity in real-time applications [39]. The theoretical approach of pyramid pooling and the u-net model combined to segmentation intestinal tumors. As to enhance the segmentation performance, so incorporated specific features of local and global T2-weighted MRI slices of 303 patient’s data in the version. Analysis revealed that the median DISA score for serous cystic neoplasms (SCN) ranged at 85. 49 ± 2. 02 and the median DISA rating on mucinous cystic neoplasms was 87. 90 ± 4. 19. Higher type identification and feature recognition were among the advantages that were enhanced. Some of the issues include their reliance on the simple MRI scans, the need for the signatures, and the verification that is done to a single laboratory despite the need for multimodal statistics and automatic strategy replication [40]. The use of deep learning (DL) applications in the diagnosis, management and monitoring of pancreatic cancer (PC). These include supervised as well as unsupervised machine, deep learning approaches in radiomics, and based neural network for PDAC, PNETs, and PCLs. The results have indicated that the DL models have enhanced the diagnostic performance and also the ability to diagnose diseases at early stages with an AUC-ROC values ranging from 0. 7 to 0. 96 in various studies. Benefits are improved accuracy in diagnosis as well as the ability to predict future outcomes; these disadvantages include variations in external validation and possible generalization difficulties that may occur [41]. Deep learning paradigm for pancreatic cancer detection using CT images that do not require large resolutions. The method used a self-supervised learning algorithm called pseudo-lesion segmentation (PS), which improved the performance of the model by generating high-quality training data Results Significant improvement in accuracy and sensitivity for both convolutional neural network (CNN) and transformer-based models when using PS demonstrated, which gave an internal accuracy of 94.3% and 95.7%, respectively Advantages include reliability a decrease on expert information, and an increase in accuracy on even smaller data sets. However, the external validation performance of the model, although robust, was lower than the internal validation, suggesting potential limitations of generalizability [42]. IDLDMS-PTC for classification of pancreatic tumor from CT images which consists of GF based pre-processing, EPO-MLT based segmentation, MobileNet based feature extraction, AE based classification and MLO based parameter optimization. It can be observed that the categorization outcome is better because of the inclusion of EPO to determine the best threshold and MLO for the correct model parameters. The main benefits include the increased accuracy of tumor detection as well as the higher speed of diagnosis. However, concerns arise when relying on neural networks which include: Increased complexity in computation and the necessity of large training data to achieve the best results. The utilization of a combination of several sophisticated techniques enhances the applied diagnostic model’s stability and accuracy [43].

The related work in this study highlights various advancements and challenges in the detection and classification of pancreatic tumors using AI and deep learning. Current AI-based CAD systems demonstrate significant promise in early detection and diagnostic accuracy, surpassing radiologists in certain metrics. Techniques such as convolutional neural networks (CNNs), deep convolutional neural networks (DCNNs), and modified models like AX-Unet and ResNeXt show improved performance in terms of sensitivity, specificity, and segmentation accuracy. However, challenges remain, including the need for large, high-quality datasets, improved result transparency, and the computational complexity of models. Techniques integrating attention mechanisms and multi-scale feature extraction enhance accuracy but also increase complexity. Despite the progress, issues such as dataset heterogeneity, model generalizability, and real-time application constraints persist. A robust, automated CAD system addressing these challenges is essential for reliable pancreatic tumor detection and classification, aiming for better clinical integration and patient outcomes.

Since current research on pancreatic tumor detection and classification is not able to produce improved performance in these areas. The heterogeneity of pancreatic tumors in terms of size, location, and form complicates the process of identifying and categorizing the many tumor types (i.e., benign, pre-malignant, malignant, and normal), which eventually impairs function. For the identification and classification of pancreatic tumors, a completely automated CAD system that is more reliable, robust, and well-balanced is thus required.

## 3. Methodology

**Fig 1:**
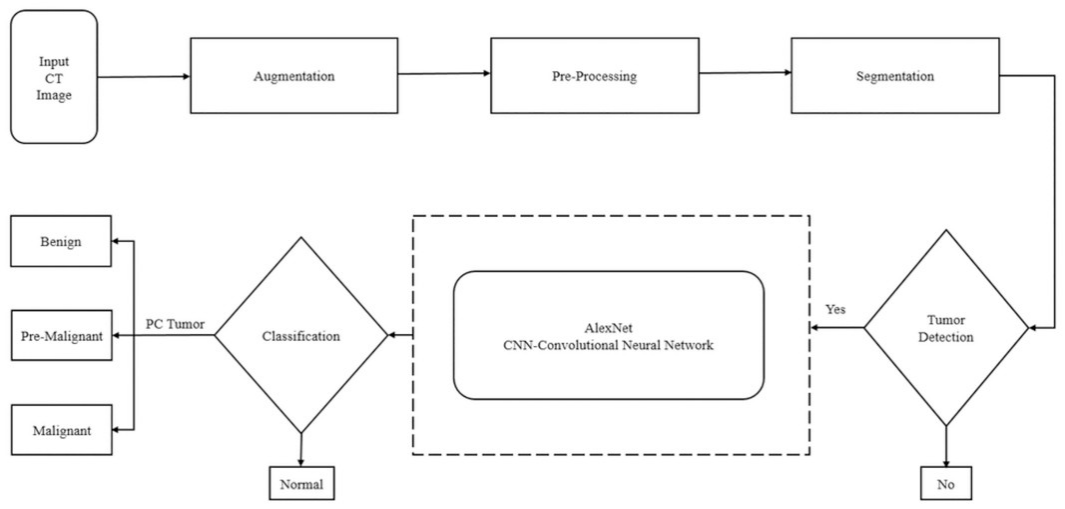
Proposed CAD system framework.

Our work presents a state-of-the-art automated CAD (computer-aided diagnosis) system to identify and classify pancreatic tumor by using CT (computed tomography) scans. Our system includes a total of four stages of preprocessing, and segmentation, (including two main stages of detection and classification). Also, we perform the five-class (i.e. normal, pancreatic tumor, benign, pre-malignant, and malignant) classification. Inspired by the success of the overall system capabilities preprocessing by Anisotropic diffusion filtering to enhance the image without blurring the edges, segmentation by watershed, optimized by feature extraction, and reduction process for the tumor detection and AlexNet-CNN 11-layer transfer learning modal for the classification of the pancreatic tumor is most reliable and robust. The system’s overall process framework is shown in Fig 1.

### 3.1 Augmentation

In augmentation, we flip the images in (vertical & horizontal) directions and randomly rotate from -30° to 45° angles to increase the dataset to make the system more robust and for regularization to avoid overfitting.

### 3.2 Pre-processing

In the Preprocessing step firstly we resized to a standard size of 227×227 pixels as input image dimension requirement. If the resulting image is in RGB format, it is converted to grayscale for further analysis based on the color channels’ luminance (perceived brightness) using Eqs (1) and (2).


gray=0.2989×R+0.5870×G+0.1140×B (1)

$$RGB\;to\;gray=\frac{R+G+B}{3}$$ (2)

Anisotropic diffusion filtering is applied to enhance the image and this preprocessing prepares the image for subsequent steps in the analysis, such as tumor detection and classification. Anisotropic diffusion filtering is employed to enhance the image with specific parameters such as the number of iterations, Time step, Conductance parameter, and Diffusion scheme regulating the details of the filtering procedure. We use anisotropic diffusion to enhance or suppress certain features in the image, contributing to feature extraction by image denoising and edge enhancement. It works by diffusing pixel values over neighboring pixels, with a diffusion rate that depends on the local gradient magnitude. This helps preserve edges while reducing noise using Eq (3). Where the image intensity changes over time $\frac{\partial I}{\partial t}$ as a function of the gradient of the image ∇*I* and the diffusion coefficient *c*. I is the input image, t is time, ∇ is the gradient operator, *c*(*x*,*y*,*t*) is the diffusion coefficient, which controls the diffusion rate based on the local image gradient. We calculate gradient magnitude by weighting factor using anisotropic diffusion to control the diffusion process based on the image gradient magnitude ∥ *∇I* ∥, where *K* is a constant as expressed in Eq (4). Furthermore, the discretized version of the equation for anisotropic diffusion filtering is implemented using finite differences to improve the image denoising and edge-preserving smoothing as expressed in Eq (5).

$$\frac{\partial I}{\partial t}=\nabla ∙\left(c\left(x,y,t\right)\nabla I\right)$$ (3)

$$c\left(x,y,t\right)=e^{-{\left(\frac{\left\|\nabla I\right\|}{K}\right)}^{2}\;}$$ (4)

$$I_{i,j\;}^{n+1}={\;I}_{i,j}^{n}+\nabla t\;(c\left(\left|{\nabla I}_{i,j}^{n}\right|\right)\;\nabla \;∙(c\left(\left|{\nabla I}_{i,j}^{n}\right|\right)\;{\nabla I}_{i,j}^{n}))\;$$ (5)

**Fig 2:**
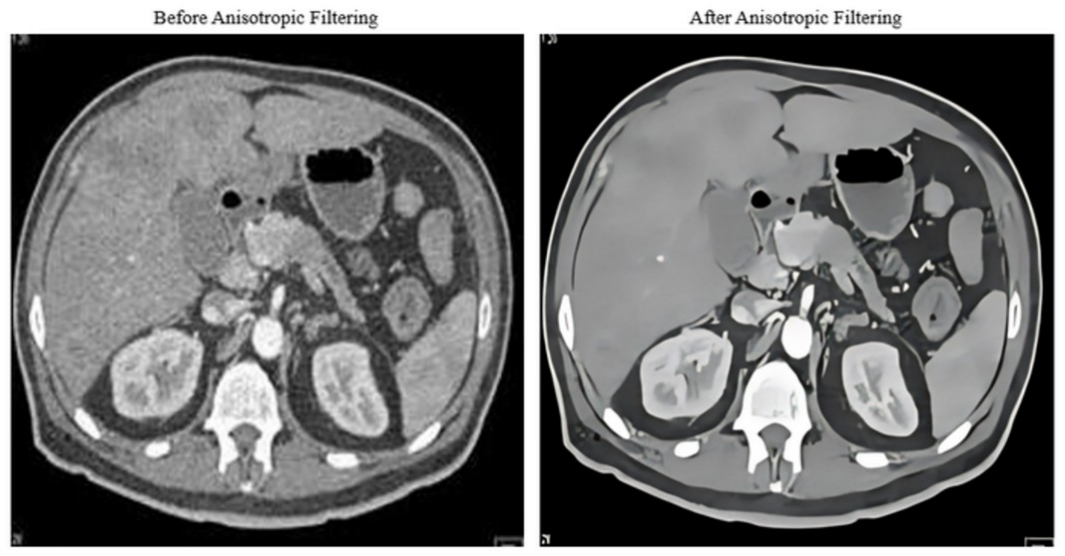
Anisotropic filtering result.

Where $I_{i,j}^{n}\;$ is the pixel value at position $\left(i,j\right)$ in the image at a time *n*. ∇t is the time step. ∇ is the discrete gradient operator, typically implemented using central differences. *c* is the diffusion coefficient function, which controls the diffusion rate based on the gradient magnitude. The superscripts n and n + 1 denote the current and updated time steps, respectively. Implementing anisotropic diffusion filtering involves iterating this update equation for each pixel in the image over multiple time steps, effectively diffusing the image’s intensity values while preserving edges shown in Fig 2.

### 3.3 Segmentation

#### Watershed

In segmentation, from the preprocessed grayscale image a binary image is created based on a threshold (*th*) as expressed in Eq (6).


binaryImage=grayImage>thresholdValue (6)

Calculates a threshold value (*th*) based on the input image (*inp*). It starts by finding the minimum and maximum pixel intensities in the input image *min* (*inp*(:)) and *max* (*inp*(:)), respectively). Then, it computes the average of these two extremes. Using thresholded image a threshold value is calculated based on the mean and maximum pixel values of the input image. This threshold sets the values of pixels above 1 and below 0. *t*_*θ*_ is a threshold value used in watershed segmentation calculated as *t*_*θ*_ = 20 this threshold is applied to determine which pixels belong to the object of interest foreground and which belong to the background. Pixels with intensity values greater than *t*_*θ*_ are considered part of the foreground, while those less than *t*_*θ*_ are considered part of the background. Finally, it adds an offset value *t*_*θ*_ to this average to obtain the final threshold value as expressed in Eq (7).


$$th=t_{\theta }+\left(\;\frac{\left(max\;\left(inp\left(:\right)\right)+\;min\;\left(inp\left(:\right)\right)\right)}{2}\right)$$ (7)

Sobel filtering is applying to the binary image to compute the gradient magnitude approximating the gradient of the image intensity function at each pixel. The gradient indicates how the intensity of the image is changing at that point and is often high at the edges of objects. The Sobel operators consist of two 3*x*3 kernels, one for detecting edges in the horizontal direction $\partial_{X}$ and the other for detecting edges in the vertical direction $\partial_{Y}$. These kernels are convolved with the image to calculate the approximations of the gradients in the X and Y directions. To compute the gradient approximation in the X direction the image is convolved with the $\partial_{X}$ kernel. Similarly, to compute the gradient approximation in the Y direction the image is convolved with the $\partial_{Y}$ kernel using Eq (8). This magnitude represents the strength of the edge at that pixel. Additionally, the direction of the edge can be calculated using Eq (9). Calculates the direction of the gradient at each pixel in the image. *atan*2 is the arctangent that returns the angle in radians between the positive x-axis and the point (X,Y) represented by the arguments $\partial_{Y}$ and $\;\partial_{X}$. The direction Ɵ is typically in the range of −*π to π*, representing the orientation of the edge at each pixel.


$$Magnitude=\sqrt{{\left(\partial_{X}I\right)}^{2\;}+\;{\left(\partial_{Y}I\right)}^{2\;}}$$ (8)

$$Ɵ=atan2\left(\frac{\left(\partial_{Y}\right)}{\;(\partial_{X})}\right)$$ (9)

**Fig 3:**
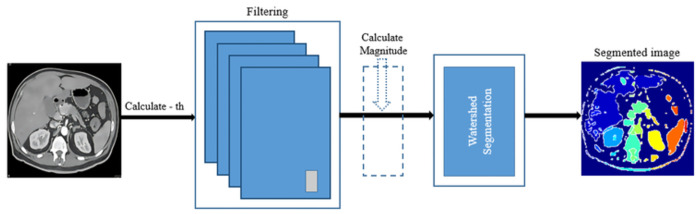
Proposed segmentation framework.

Sobel filter highlights edges by computing the gradient magnitude of the image. This can help to delineate objects or regions in the image that have different intensities or textures, making it easier for the watershed algorithm to identify and separate these regions. After applying the Sobel filter, we can use the watershed algorithm to segment the image based on the gradient magnitude. The watershed algorithm treats the gradient magnitude as a topographic surface, where the intensity values represent the heights of the surface. The algorithm then "floods" the surface from markers (seed points) and delineates regions where the "water" segmentation boundaries from different markers meet. Overall, using Sobel filtering before watershed segmentation can help improve the segmentation results, especially when dealing with images with complex textures or intensity variations. After Compute the gradient magnitude of the image to highlight the edges used Sobel edge detection filters. Let’s denote this gradient magnitude image as M. generates markers in the image. These markers can be manually defined or obtained using techniques thresholding. Treat the gradient magnitude image M as a topographic surface and consider the markers as sources of "water". Imagine flooding the surface from these markers. The water is initially fill up basins (regions) around the markers. As the water level rises, regions from different markers is start to merge. To prevent this, we can use a method to identify and mark the points where waters from different basins meet. These points are often called watershed lines or lines of separation. Finally, the watershed lines form the boundaries of the segmented regions. The regions enclosed by these lines are the segmented objects or regions. The result of the watershed segmentation (*L*) provides a partitioning of the image into distinct regions, enabling further analysis or classification of objects within the image using Eq (10) and shows in Fig 3.


$$L=watershed\left(magnitude\right)$$ (10)

Watershed segmentation is a technique used in image processing to segment regions based on the topography of the image. It can be useful for separating objects in images, such as tumors in medical images.

#### U-Net

Defined the directories for the tumor and normal images. These directories contained the CT scan images that were used for training and validation. Image datastores were created for the tumor and normal images separately. Each image datastore held the file paths and labels for the respective images. The labels were manually assigned, with "tumor" for tumor images and "normal" for normal images. The tumor and normal image datastores were then combined into a single image datastore. Directories for the pixel label data corresponding to the tumor and normal images were defined. Pixel label datastores were created for these labels, with class names "background" and "tumor" and corresponding label IDs 0 and 1. These pixel label datastores were combined into a single pixel label datastore. The combined image and pixel label datastores were partitioned into training and validation sets with a ratio of 70% for training and 30% for validation. This was done using a custom partition function. The U-Net architecture was defined with an input image size of 227x227 and a single channel. The number of classes was set to 2, representing background and tumor. Data augmentation was optionally applied to the training data to improve model generalization. This included random reflections and translations.

The training process for the U-Net model was carefully configured to ensure optimal performance. We used the Stochastic Gradient Descent with Momentum (SGDM) as our optimizer, which helps accelerate gradients vectors in the right directions, thus leading to faster converging. An initial learning rate of 1e-4 was set to control the step size during the update of model parameters, ensuring gradual and stable learning. The execution environment was set to auto-select between CPU and GPU to leverage the available hardware for efficient training. The training was conducted over 20 epochs, with each epoch representing a complete pass through the entire training dataset. A mini-batch size of 16 was used, allowing the model to update its parameters more frequently and effectively. Validation was performed every 10 iterations to monitor the model’s performance on the validation dataset and to prevent overfitting. The data was shuffled at every epoch to ensure that the model was exposed to a diverse set of samples in each epoch, promoting better generalization. The validation dataset was explicitly provided to evaluate the model’s performance on unseen data, giving insights into how well the model might perform in real-world scenarios. Training progress was plotted to visually monitor the training and validation loss, facilitating easier debugging and adjustment of training parameters if needed. Finally, the verbose output was set to false, ensuring a cleaner display by suppressing detailed outputs during training.

The U-Net model was trained using the defined architecture, training options, and augmented training data. The training process involved iteratively updating the model weights to minimize the loss on the training data while monitoring performance on the validation data. This comprehensive approach ensured that the U-Net model was effectively trained to segment pancreatic tumors from CT scan images, facilitating accurate and efficient tumor detection and classification in our research.

### 3.4 Feature extraction and reduction

Furthermore, before the detection and classification we perform morphological refinement refers to the use of morphological operations, to improve the image results. After performing the initial segmentation of the image to separate objects from the background, morphological refinement can be applied to clean up the segmentation result and improve object delineation. This can involve removing small isolated regions (noise), filling in gaps or holes in segmented objects, and smoothing object boundaries. Applying morphological operations strategically improves the accuracy and reliability of subsequent image results. The resulting image highlights edges and other high-frequency features to help for further feature extraction. Morphological operations, such as dilation and erosion, can modify the shape and structure of objects in the image, potentially contributing to feature extraction or reduction. Then we use Erosion to remove pixels at the boundaries of objects in the image to shrink the size of objects using Eq (11). Where (*A*) is the input of the binary image and $B_{z\;}$ is the structuring element centered at z. The opening is an erosion followed by a dilation is used to remove small objects or noise from the image using Eq (12). Then Dilation adds pixels to the boundaries of objects in the image to expand the size of objects closing is a dilation followed by erosion helps to fill the small gaps or holes in objects using Eqs (13) and (14).


$$E\left(A\right)=\left\{z\;\in \;z^{2}\;|\;B_{z\;}\subseteq A\right\}$$ (11)

$$O\left(A\right)=D\left(E\left(A\right)\right)$$ (12)

$$D\left(A\right)=\left\{z\;\in \;z^{2}\;|\;B_{z\;}\cap A\;\neq \;\emptyset \right\}$$ (13)

$$C\left(A\right)=E\left(D\left(A\right)\right)$$ (14)

After performing morphological refinement on an image, the next step involves using the Gray-Level Co-occurrence Matrix for texture analysis and extracting texture features from images. GLCM is computed by analyzing the spatial relationship of pixel intensities in image after morphological refinement. It counts the occurrences of pixel pairs with specific intensity values and spatial relationships. From the GLCM, various texture features can be extracted, such as contrast, correlation, energy, and homogeneity. These features describe different aspects of texture, such as the distribution of intensity levels and the regularity of patterns. The extracted texture features can be analyzed to characterize the texture properties of regions in the image. This analysis can help in distinguishing between different materials or objects based on their texture characteristics. The texture features extracted from GLCM are used as input for detection and classification algorithms. These algorithms can automatically classify or segment regions in the image based on their texture properties. Further improving, we extract texture features by the gray level co-occurrence matrix using the gray comatrix for better texture analysis and feature extraction. Texture features derived from the gray level co-occurrence matrix help us as input features to classify the textures or objects in the image.

Computing the Gray-Level Co-occurrence Matrix (GLCM) involves analyzing the spatial relationship of pixel intensities in image. Let’s the image as *I* with intensity values ranging from 0 to *L*–1, where ***L*** is the number of gray levels. The GLCM is a ***L*** *×* ***L*** matrix ***P*** where ***P***(***i***,***j***) represents the number of times a pixel with intensity ***i*** occurs at a specified spatial relationship to a pixel with intensity ***j***. The spatial relationship can be defined by the distance between two pixels and the direction (angle) relative to each other. For simplicity, let’s consider a single direction (0 degrees, which is typically horizontal) and a distance of 1 pixel. The GLCM calculation for this direction and distance expressed in Eq (15). Where N and M are the dimensions of image I. This formula counts the occurrences of pixel pairs with intensities i and j at a distance of 1 pixel horizontally. In practice, the GLCM is often normalized to obtain a probability matrix ′P′. This normalization ensures that the values in the GLCM represent probabilities of occurrence rather than raw counts.


$$P\left(i,j\right)=\sum \begin{array}{c} N-1\\ x=0 \end{array}\sum \begin{array}{c} M-1\\ y=0 \end{array}\left\{\begin{array}{cc} \begin{array}{c} 1\\ 0 \end{array} & \begin{array}{c} if\;I\left(x,y\right)=i\;and\;I\;\left(x+1,y\right)=j\\ otherwise \end{array} \end{array}\right\}$$ (15)

$$P\left(i,j\right)=\frac{P\left(i,j\right)}{\sum \begin{array}{c} L-1\\ i=0 \end{array}\sum \begin{array}{c} L-1\\ j=0 \end{array}\;P\left(i,j\right)}$$

**Fig 4:**
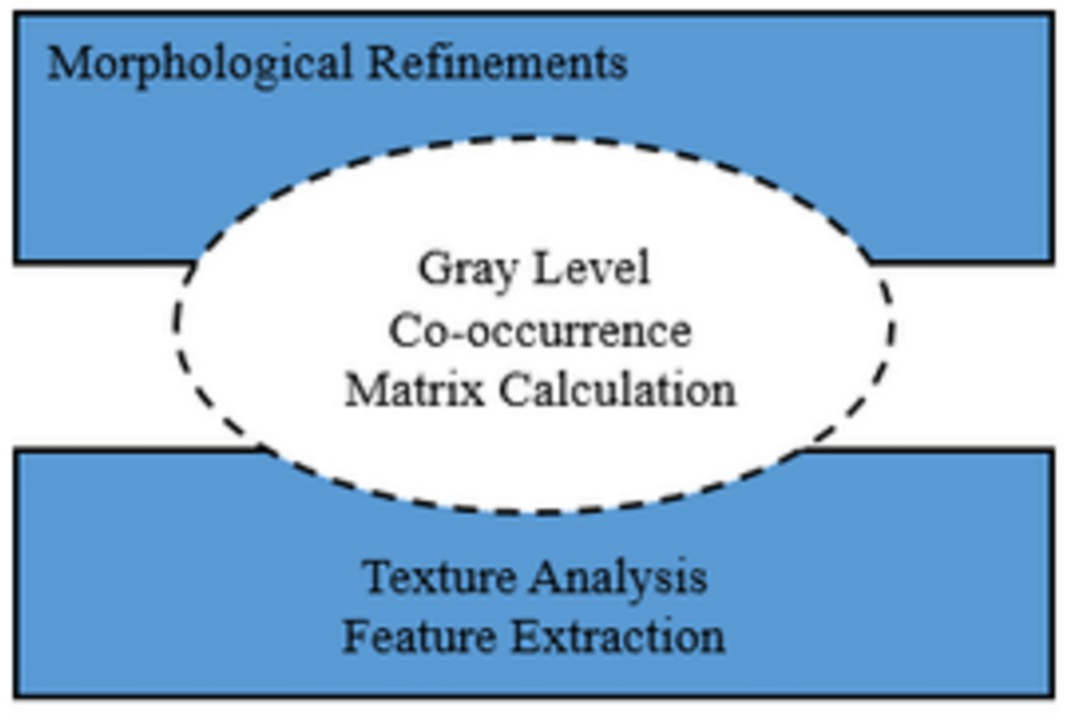
Feature extraction & reduction framework.

This Fig 4. Represents the flow of processing steps from the original input image to various image enhancement and feature extraction processes, including morphological refinements, calculation of the gray level co-occurrence matrix, and texture analysis for feature extraction. Each step contributes to enhancing certain features in the image or extracting relevant information for further analysis and classification. Various image statistics are expressed in Table 1. Such as contrast, mean, energy, entropy, correlation, standard deviation, RMS, smoothness, perimeter, and centroid (x,y) help in identify the tumor. These statistics could be considered as features for detection and classification.

**Table 1:** Features and formulas.

Features	Formulas
Contrast	*tumorcontrast* = *max*(*tumor*(:)) − *min*(*tumor*(:))
Mean	*meantumor = mean(tumor*(:))
Energy	*glcms* = *graycomatrix*(*tumor*); *stats* = *graycoprops*(*glcms*); *eng* = *num*2*str*(*stats*.*Energy*);
Entropy	*tumorentopy* = *entropy*(*tumor*);
Correlation	*R* = *corr*2(*I*,*J*); (*where I and J are matrices*)
SD	*sdtumor* = *stdfilt*(*tumor*(:))
RMS	*rmstumor* = *rms*(*x*); (*where x is a vector*)
Smoothness	*smoothnessMetric* = *mean*2(*sdImage*); (*where sdImage is the standard deviation image*)
Perimeter	detected_tumor_perimeter1 = detected_tumor_perimeter*0.26458333; (assuming detected_tumor_perimeter is in pixels, converting to mm)
Centroid (x,y)	*detected_tumor_centroid* = *region_measure*.*Centroid*

### 3.5 Detection of tumor

The tumor detection and localization process involves region labeling and subsequent analysis of labeled regions. The visualization techniques include displaying the binary tumor image, drawing bounding boxes around the tumor, and outlining the tumor boundaries. These steps collectively contribute to the identification and visualization of the detected tumor region in the medical image. The original preprocessed image is thresholded based on a dynamic threshold value and pixels above the threshold are set to 1, indicating a potential region of interest (ROI) or tumor, while pixels below the threshold are set to 0 using Eq (16). Then Morphological operations are applied to the thresholded image sout to enhance the tumor region. The system uses morphological labeling to label connected components (regions) in the binary image and these operations modify the binary image to enhance or suppress features using Eqs (17) and ((18).


$$BinaryImage\left(x,y\right)=\left\{\begin{array}{c} 1,\\ 0, \end{array}\begin{array}{c} \;ifImage\left(x,y\right)>Threshold\\ Otherwise \end{array}\right\}$$ (16)

$$sout\left(x,y\right)=\left\{\begin{array}{c} 1,\\ 0, \end{array}\begin{array}{c} \;ifinp\left(x,y\right)>th\\ otherwise \end{array}\right\}$$ (17)

$$erosion\left(A,B\right)\left(x,y\right)=\cap_{\left(i,j\right)\in B}A\left(x+i,y+j\right)$$ (18)

Statistics like solidity, area, and bounding box are calculated for each labeled region using region props. The density array is created to store the solidity values of the labeled regions. High-dense detected tumor area is a logical array indicating regions with high density (solidity > 0.6) and the maximum area among high-density regions is considered as the detected tumor area as expressed in Eq (19).


high_dense_detected_tumorarea=solidity>0.6 (19)

**Fig 5:**
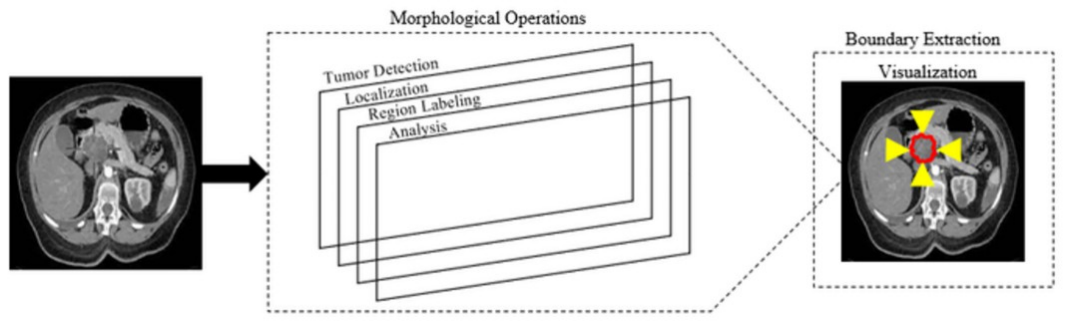
Proposed tumor detection & localization framework.

The label corresponding to this maximum area is identified as the tumor label. A image (tumor) is created by selecting only the pixels belonging to the tumor label. The whole process architecture of tumor detection and localization is shown in Fig 5.

#### 3.5.1 Tumor localization

The system checks if the detected tumor area (max detected tumor area) is greater than a threshold. And the tumor area is significant, it displays the binary tumor image (tumor). The bounding box (wanted Box) of the detected tumor region is obtained from the region properties. This bounding box is visualized by drawing a yellow rectangle on the original preprocessed image. A dilation operation is applied to the tumor region (eroded Image) to expand it. The tumor outline (tumor outline) is obtained by subtracting the eroded image from the original tumor image as expressed in Eq (20). Applying dilation to the tumor region (eroded Image) to expand and join broken parts of the tumor region, filling gaps to restore the original shape and size of the tumor region using Eq (21)). Obtaining the tumor outline (tumor outline) by subtracting the eroded image from the original tumor image using Eq (22).


$$Original\;image\left(x,y\right)=\left\{\begin{array}{c} yellow,\\ original\_image\left(x,y\right), \end{array}\begin{array}{c} \;if\left(x,y\right)\;is\;within\;wantedBox\\ otherwise \end{array}\right\}$$ (20)

$$dilated\;Image=imdilate\left(tumor,SE\right)$$ (21)

tumorOutline=tumor-erodedImage (22)

**Fig 6:**
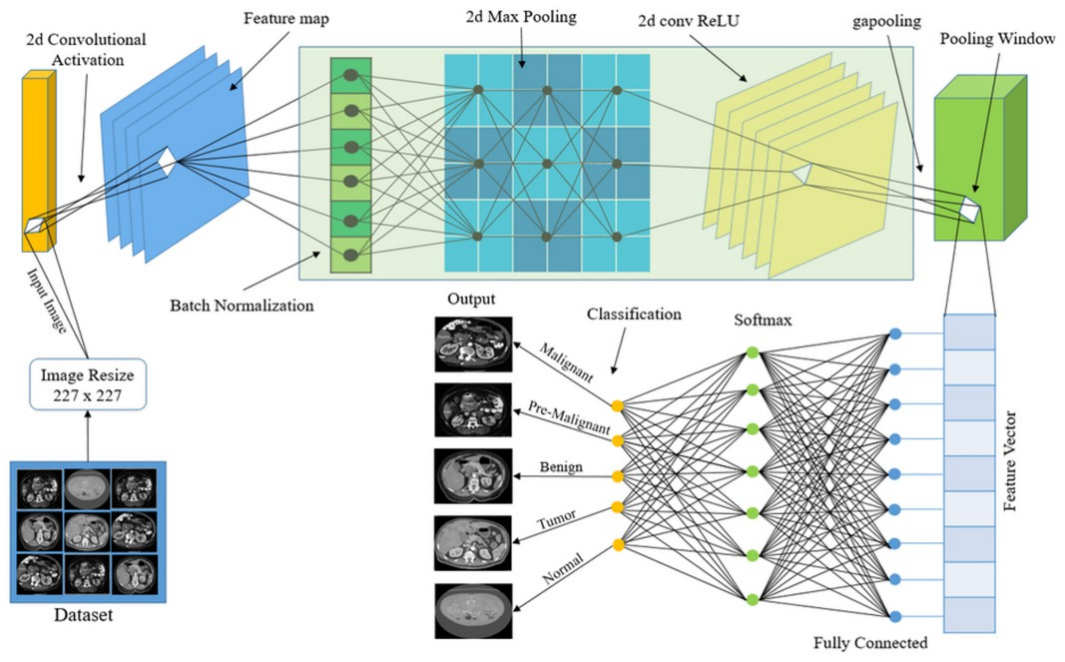
Proposed 11-layer AlexNet-CNN framework for classification.

The outlined tumor is displayed and boundaries are extracted from the resized binary tumor image. The boundaries are a cell array, each element containing a set of (x, y) coordinates forming a boundary. Then plots these boundaries on the image with a yellow line are shown in Fig 6.

### 3.6 Proposed AlexNet classification modal

In Fig 6. And Table 2. The proposed AlexNet Modal is based on reduced 11 layers and 10 connections. The process commences with the Image Input layer, which represents the image input. Subsequently, the 2D Convolutional Layer with 96 filters and an 11x11 filter size convolves the input gray image, generating a series of feature maps with the ReLU (Rectified Linear Unit) activation function. Following this, the Batch increases training speed and stability, and the normalization layer normalizes the output of the previous layer. Then, by choosing the largest value inside localized regions, the 2D Max Pooling layer decreases the dimensionality of the feature maps. Next, another 2D Convolutional Layer with 32 filters and a 3x3 filter size applies a convolution operation to the output. This process is repeated with an additional 2D Convolutional Layer that utilizes 32 filters of size 3x3. Subsequently, the 2D Global Average Pooling (GAP) layer condenses the feature maps to a single value by averaging all values within each map, effectively reducing the number of parameters. Following this, a Max Pooling layer conducts further pooling akin to previous operations. Using a set of weights (2x32) and biases (2x1), the 2D Fully Connected layer creates connections between each neuron in the previous layer and those in the next layer. As the last classification layer in the network, the Softmax Probability layer uses the Softmax function to compute class probabilities based on the output of the fully connected layer that came before it.

**Table 2:** Proposed AlexNet-CNN 11 layers details.

Layers	No. of filters	Filter size	Weights	Bias
Input-Image Layer				
2d-Convolutional layer	96	11, 11	11x11x1x96 single	1x1x96 single
Batch Normalization				
2d-Max Pooling				
2d-Convolutional layer	32	3, 3	3x3x96x32 single	1x1x32 single
2d-Convolutional layer	32	3, 3	3x3x32x32 single	1x1x32 single
2d gapool layer				
Maxpool-layer				
2d Fully Connected			2x32 single	2x1 single
Softmax Prob Layer				
Classification output				

An 80/20 approach is used to divide the dataset into training and validation sets. This is essential for evaluating how well the model performs on data that it hasn’t encountered during training, which aids in determining how well it can generalize. The images in the dataset are resized to a standardized input size. This step ensures consistency in the dimensions of the images, which is necessary for feeding them into the neural network. During the data augmentation step, each image in the training dataset undergoes random rotations. The rotation is applied within a specified range, from -30 degrees to 45 degrees. This random rotation introduces variability to the training set, ensuring that the model becomes robust to different orientations of objects in the images. As the optimization algorithm, stochastic gradient descent with momentum, or sgdm, is used. The model’s parameters are iteratively adjusted by this approach to minimize the training loss, the initial learning rate is set to 0.001, and training is carried out for a maximum of 60 epochs, which is represented by Eq (23).


$$v_{t+1}=\beta ∙v_{t}+\left(1-\beta \right)∙\nabla J\left(\theta_{t}\right)$$ (23)

$$\theta_{t+1}=\theta_{t}-\alpha ∙\left(v_{t+1}\right)$$

The number of times the model processes the complete dataset is indicated by epochs, and the learning rate sets the step size for parameter changes. 256 sample mini-batches are used for training. Mini-batch training updates the weights using a part of the dataset rather than the full dataset, assisting in the efficient optimization of the model. Every epoch, data is shuffled to avoid the machine learning the training sample order by heart. Every 30 epochs, validation is carried out, enabling tracking of the model’s performance on a different validation set. An input layer that specifies the dimensions of the input images is where the neural network begins. Hierarchical features in the photos are captured by convolutional layers that use varying numbers and sizes of filters. Where *Y* is the output feature map, *X* is the input image is the filter, *b* is the bias, and *M* and *N* are the dimensions of the filter as expressed in Eq (24). To normalize the activations in intermediary layers, batch normalization is applied, which speed up training and enhance generalization. In the given equation, *X* represents the input, *μ* stands for the mean, *σ*2 for the variance, ϵ is a tiny constant for numerical stability, *γ* for scaling, *β* for shifting, and *y* for the outputs as expressed in Eq (25). Max-pooling layers increase the receptive field and decrease computing complexity by down-sampling the spatial dimensions. Where *s* is the stride, *X* is the input feature map, and *Y* is the down-sampled output as expressed in Eq (26).


$$Y\left[i,j\right]=\sum \begin{array}{c} M-1\\ m=0 \end{array}\sum \begin{array}{c} N-1\\ n=0 \end{array}X\left[i+m,j+n\right]\times W\left[m,n\right]+b$$ (24)

$$\hat{x}=\frac{x-\mu }{\sqrt{\sigma^{2}+\varepsilon }}$$ (25)

$$Scaling\;and\;shifting:y=\gamma \hat{x}+\beta$$

$$Down-Sampling:Y\left[i,j\right]={max}_{m,n}\;X\left[i\times s+m,j\times s+n\right]$$ (26)

The spatial dimensions are then reduced to one value per feature by the global average pooling layer, which computes the average of each feature map. This contributes to the network’s increased resilience to input spatial translations. Where *M* and *N* are the feature map’s spatial dimensions as stated in the equation, *Y* is the output, and *X* is the input feature map as expressed in Eq (27). Finally, the final classification is performed using dense, completely linked layers. To create predictions, these layers incorporate the characteristics that convolutional layers have learned. *W* is the weight matrix, *X* is the input vector, *b* is the bias vector, and *ϕ* is the activation function (e.g., ReLU), with *Y* serving as the output. Every neuron’s output is subjected, element by element, to the ReLU activation function. In a fully connected layer, if *x* is the neuron’s input, then y is the neuron’s output following the ReLU application as expressed in Eq (28). This indicates that the output *y* is equal *x* if the input *x* is higher than or equal to 0. Should *x* be negative, the result is zero. By doing this, the network gains non-linearity, which enables it to recognize intricate patterns in the input. The last layer uses a softmax activation function to transform raw predictions into class probabilities. The predicted class is assigned by the classification layer according to the highest likelihood. Where ***x***_***i***_ is the class *i* raw output value, and these values are transformed into probabilities by the softmax function as expressed in Eq (29).


$$Y\left[k\right]=\frac{1}{M\times N}\sum \begin{array}{c} M-1\\ i=0 \end{array}\sum \begin{array}{c} N-1\\ j=0 \end{array}X\left[i,j,k\right]$$ (27)

$$output\;calculation:Y=\emptyset \left(W∙X+b\right)$$ (28)

$$ReLU:\;y=max\left(0,x\right)$$

$$calculation:softmax\left(x_{i}\right)=\frac{e^{x_{i}}}{\sum_{j}e^{x_{j}}}$$ (29)

The model is trained using the specified options and the augmented training dataset. The network’s weights are repeatedly changed during training in order to reduce the discrepancy between anticipated and real labels. Optionally, the training progress is visualized using plots that show the evolution of training and validation metrics over epochs. This helps in monitoring whether the model is learning effectively or if adjustments to the training setup are needed. After the completion of training, a fully trained neural network is obtained. This model is capable of making predictions on new, unseen data based on the patterns it learned during training.

All things considered, we well-trained the reduced 11-layer AlexNet is a deep convolutional neural network made for classification tasks involving images. It is composed of several convolutions, pooling, and fully linked layers, with a softmax layer for classification coming after.

## 4. Experiment, results, and evaluation

This section provides an in-depth analysis of the Computer-aided diagnosis (CAD) system based on the findings of many tests intended to evaluate the suggested methodology’s effectiveness in terms of detection and classification. Furthermore, this section includes details on the dataset that was utilized to identify and categorize pancreatic tumors. The details of the experimental setups, system specifications, and protocols are mentioned in Table 3.

**Table 3:** Tools requirements and system specification.

Name	Details
GPU of the computer system	4GB NVIDIA-Quadro P600
CPU of the computer system	Intel(R)-Core(TM), i5-8300H, 2.30GHz
NVMe	512GB SX6000PNP
HDD	1TB disk drive
RAM	16GB
Implementation Tool	Paid MATLAB R2023b
Window	Window 11Pro, 64 bit

### 4.1 Dataset

**Fig 7:**
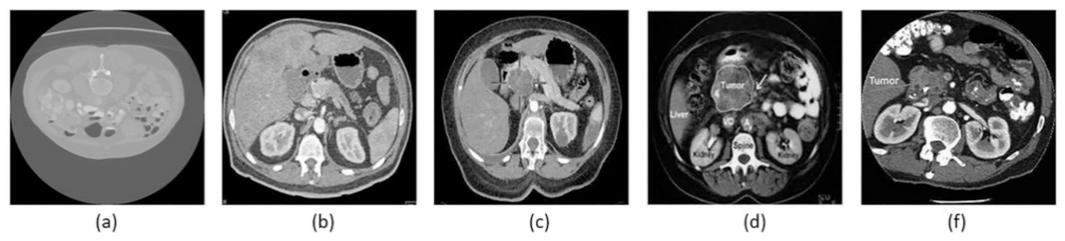
Dataset of CT images for pancreatic cancer.

To conduct this research, we used a publicly available CT scans dataset. Gathered pancreatic tumor CT scans from patients. There are 1411 pancreatic CTs images in total, including the 646 normal images and 765 pancreatic tumor images (benign, pre-malignant, and malignant) Fig 7. Shows examples of the various classes (a to f) of pancreatic tumor images. The image is a 2D volume with 512 × 512. The dataset format is available online in.jpg format. In this study, 1129 CT images (80%) were used for training, while 282 were used for testing (20%). In Table 4. Contains detailed information about the CTs dataset, such as the number of images and class labels for each type of pancreatic cancer (normal, pancreatic tumor (benign, pre-malignant, and malignant)) as shown in Fig 7.

**Table 4:** Pancreatic tumor CTs images dataset details.

Class	Images	Class label	Testing data	Training data
Normal	646	1	129	517
Pancreatic Tumor (benign, pre-malignant, and malignant)	765	2	153	612
Total	1411		282	1129

### 4.2 CAD system design and implementation (GUI)

**Fig 8:**
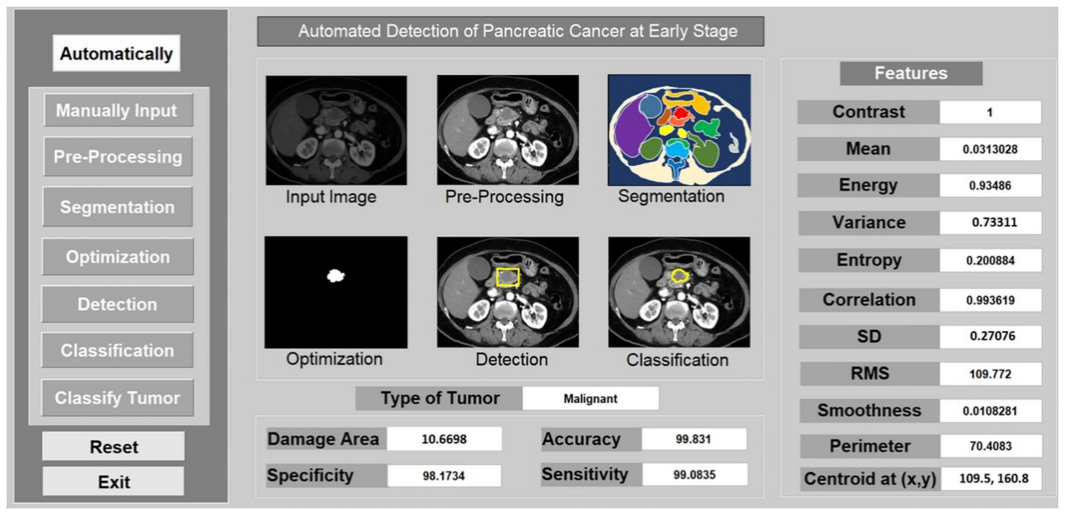
Proposed GUI-based CAD system for PC Tumor.

In Fig 8. The GUI (graphical user interface) of the CAD (computer-aided diagnosis) system meets all the design standards of user experience. The system interface is fully loaded with processing functional buttons including (Automatic, and manual input as pre-processing, segmentation, optimization, detection, classification to classify tumor and reset/exit), six axes for showing image results, and feature parameters including (contrast, mean, energy, variance, entropy, correlation, standard deviation, RMS, smoothness, perimeter, and centroid of x,y). The system application interface also shows the type of tumor, damage area, and performance matrices of accuracy, sensitivity, and specificity.

#### Watershed vs. U-Net segmentation

Here is the detailed comparison between watershed vs. U-Net segmentation in the case of our research study:

Table 5 presents a detailed comparison of the experimental results obtained using watershed segmentation and U-Net segmentation methods. The data in this table highlights our own findings, with accuracy, segmentation process, and feature extraction performance analyzed for each method.

**Table 5:** Comparative results of watershed vs. U-Net segmentation in pancreatic tumor detection.

No.		Watershed Segmentation	U-Net Segmentation
1.	Segmentation		
	Process	Binary Image Creation and Edge Highlighting: The watershed method uses the preprocessed binary image and the highlighted edges to segment the tumor region effectively. The method’s reliance on clear boundaries aids in accurate segmentation [7, 26].Morphological Operations: Post-segmentation, morphological operations refine the tumor’s geometric structure, enhancing the segmentation quality and leading to a high accuracy of 99.5%. Utilizes markers and morphological operations to define regions of interest. Treats the image as a topographic map, effectively segmenting areas with clear boundaries.	Segmentation Process: U-Net has convolutional network followed by a de-convolutional network for segmenting the image. Despite its sophisticated architecture, U-Net struggled to achieve comparable accuracy (92. 1%) due to: Despite its sophisticated architecture, U-Net struggled to achieve comparable accuracy (92. 1%) due to:Complexity of Tumor Shapes and Sizes: U-Net may fail to request functional details such as the deviation in structure boundaries, size, and position from the training it receives.Edge Preservation: While containing a learning element, as seen earlier, it is paramount to acknowledge that the U-Net algorithm does not sharpen edges as effectively as the watershed does, which results in a lesser degree of segmentation accuracy [37]. It employs an encoder-decoder network with short connections to provide training features of different levels of abstraction. It depends on the presence of training data to provide segmentation of the underlying patterns.
	Advantages	Ideal for splitting areas that are easily separable or areas that are of conspicuous different shapes. Easily applicable because the boundary of a character can be easily detected, and highly accurate, achieving an accuracy of 99. 5% in our experiment.	It is particularly effective in apprehending context and small components of that scenario. Can generalize well when have enough and varying training examples available to work on.
	Challenges	Noise level is a major factor which needs to be controlled as well as the location where the markers are initially placed. This may result into over-segmentation especially in the case where the markers are not well developed [39].	It means that the effectiveness of the performance is highly correlated with the quality and amount of training data provided. In our case, it achieved 92 per cent. In regards to performance, the following were the findings: 1. 1% of accuracy that would clearly show that there are some problems with the training dataset or on the network structure.
2.	Post-Segmentation Processing		
		Procedural methods which perform morphological operations (erosion, dilation) and eliminate irrelevant small regions. In identifying different regions of a tumor, connected component labeling may be of use.	Generally, it does not need many subsequent steps to be refined as it can learn directly and predict accurate segmentations. Thus to the problem we still might owe the small refinements or smoothing techniques.
3.	Feature Extraction and Detection		
		Feature Extraction and Analysis: The convergence of the method of watershed segmentation of the images as well as morphological operations in the vicinity of the images assist in the correct identification and location of the tumor area enhanced by the gray-level co-occurrence matrix for the extraction of the feature of texture [35, 36]. Thereby enhancing the accuracy of detection where the method labels connected components appropriately and selects the region of highest density of the desire features (99. 64%). It extracts features based on defined regions and subregions such as the region of interest or area of interest. By calculating and analyzing the density of connected components, it identifies the major connected area where the primary tumour is located.	Feature Extraction and Analysis: With the added feature maps, U-Net croaks tumor directly but may not have the ‘O’ and ‘G’ of watershed to preserve edges and grow region of interests, hence poor detection might be realized [26, 27]. Deploy the segmented regions based on the learned segmentation. If the segmentation is not accurate, it may be necessary to perform further steps to extract relevant features based on clustering requirements.
4.	Classification		
		Refined Segmentation for Classification: Huge quality of segmentation, which was implemented with the help of the watershed procedure, allows the recognizer to classify the regions of the detected tumor with a help of the minimized 11-layer AckNet model correctly. In deep learning, compared to the models trained on large nuclei images, the clear demarcation of tumor boundaries results in better SE separation and hence better feature extraction for classification, achieving an accuracy of 98%. 72% and of area under receiver-operating characteristic (AUC) was found to be 0. 9979 [37, 38].	Segmentation Output for Classification: The use of low-quality segmentation from the U-Net impacts the subsequent classification phase. A potential drawback of the less sharp segmentation boundaries may be the additional noise involved in the task of feature extraction which might result in the lower classification rates as compared to the watershed method [37].
	Both Methods	Use a reduced 11-layer AlexNet model to classify the detected tumor regions. The effectiveness of classification depends on the accuracy and precision of the segmentation stage.	
5.	Overall System Performance		
		Average Processing Time: The watershed method’s combination of preprocessing, segmentation, and detection stages results in an overall system average processing time of 1.51 seconds, demonstrating its efficiency.	Processing Time and Performance: Thereby, revealing that although the U-Net network is rather effective at solving multiple problems, it did not demonstrate such efficiency and accuracy in this case. It can also be the reason behind a long processing time and lower performance rates in our particular approach and methodology due to training and tuning additional complexities.
	Overall Recommendation Watershed segmentation is highly compatible with our method due to its ability to generate high accuracy and good control of intestinal tumors with clear boundaries. This makes our system a more reliable method for the next step of identification and classification. The methodological alignment of the watershed approaches to our specific requirements achieved high results, demonstrating consistency and efficiency in our system		

### 4.3 Detection results and comparison

The performance of the detector has been evaluated using evaluation metrics. F1 score, AUC matrix, accuracy, sensitivity, specificity, precision, recall, and F1 score have all been used to evaluate the detection performance.

**Fig 9:**
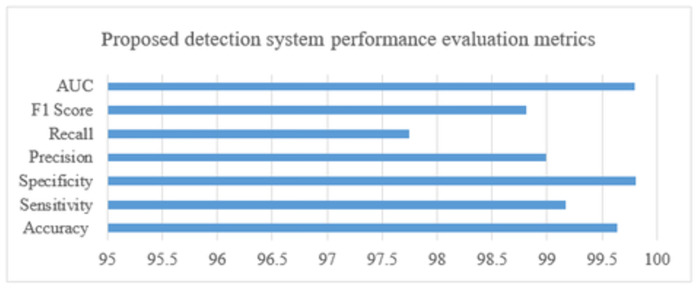
System performance metrics for tumor detection.

In the above Fig 9. Performance metrics for the proposed detection system. We were able to detect pancreatic tumors with an accuracy of 99.64, sensitivity of 99.17, specificity of 99.81, precision of 98.99, recall of 97.75, F1 score of 98.81, and AUC of 0.998, which is an overall good performance result. The suggested detection model was trained and tested using the public dataset.

**Fig 10:**
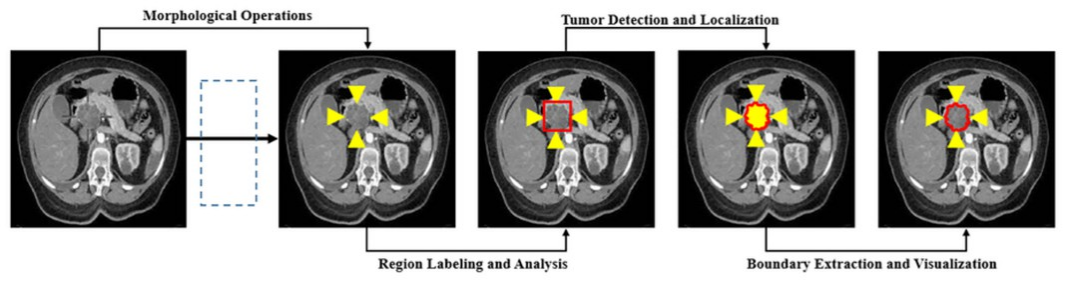
Pancreatic tumor detection results.

In Fig 10. The detection results show how precisely and accurately our system detects the tumor in the pancreas. Firstly the image given to the system, our proposed system applies thresholding using the pixel value for segmented images a particle region, contains objects under inspection and separates it from the background region. Then the morphological operations apply and label the region by analyzing it more precisely. After that detect the affected area of the pancreas and localize the tumor. And show the tumor area by boundary extraction and visualize it for further classification.

**Fig 11:**
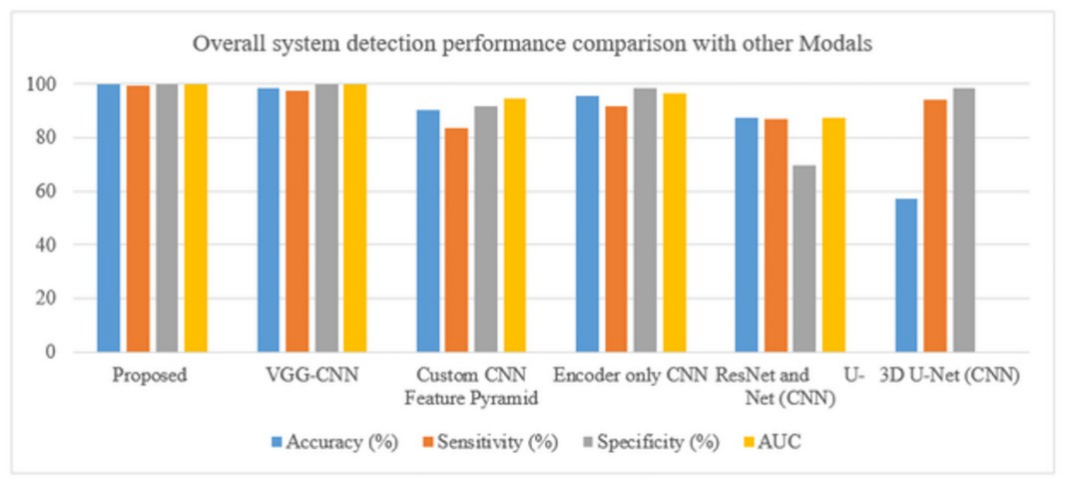
Comparison chart of performance metrics with other models for tumor detection.

**Fig 12:**
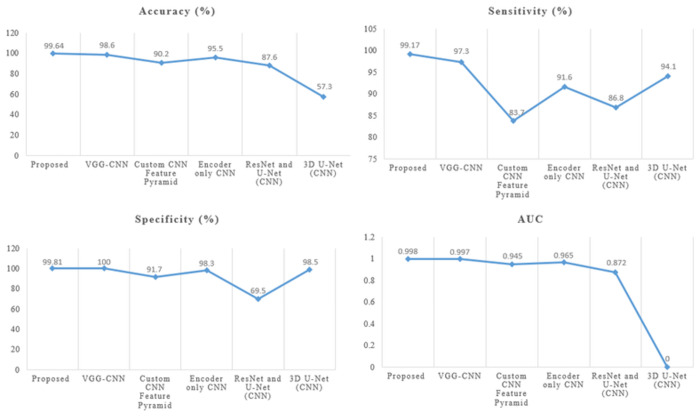
Comparison graph of performance evaluation with other models for tumor detection.

In Table 6. To assess the overall worth of our system, we compare it with alternative models. Using the dataset of 1411 CT images, we conducted this experiment and obtained an overall 99.64 detection accuracy, 99.17 sensitivity, 99.81 specificity, and 0998 area under the ROC curve (AUC). In [23] VGG-CNN the CT images of 320 patients of pancreatic cancer are used to experiment to achieve the results of accuracy is 98.6, sensitivity of 97.30, specificity of 100, and area under the curve of 0.997. [24] 2890 CT images are used in the custom CNN feature pyramid and the results of accuracy is 90.2, sensitivity of 83.7, specificity of 91.7, and area under the curve is 0.945. [25] Using the CT scans of 222 PDAC patients and 190 controls, encoder-only CNN produced results including 95.5 percent accuracy, 91.6 percent sensitivity, 98.3 percent specificity, and 0.965 percent area under the curve. A combination of [26] ResNet and U-Net CNN used the images of 319 pancreatic cancer patients and got the accuracy results of 87.6, sensitivity of 86.8, specificity of 69.5, and area under the curve is 0.872. in [27] 3D U-Net CNN 136 PDAC CT images and 303 controls are used to perform the experiment and achieve the results of accuracy is 57.3, sensitivity of 94.1, specificity of 98.5, and area under the curve is not available for this model as shown in the above table and graphically represented in below Figs 11 and 12.

**Table 6:** Detection performance metrics comparison of proposed and other models.

Model	Dataset	Accuracy (%)	Sensitivity (%)	Specificity (%)	AUC
Proposed	1411 CT images	99.64	99.17	99.81	0.998
VGG-CNN [23]	370 Pancreatic cancer 320 Control	98.6	97.3	100	0.997
Custom CNN Feature Pyramid [24]	2890 CT images	90.2	83.7	91.7	0.945
Encoder only CNN [25]	222 PDAC 190 Control	95.5	91.6	98.3	0.965
ResNet and U-Net (CNN) [26]	319 patients	87.6	86.8	69.5	0.872
3D U-Net (CNN) [27]	136 PDAC 303 Control	57.3	94.1	98.5	N.A.

### 4.4 Classification results and comparison

The classification performance has been evaluated using evaluation measures. Accuracy, sensitivity, specificity, precision, recall, F1 score, and AUC matrix have all been used to assess the classification performance outcomes.

**Fig 13:**
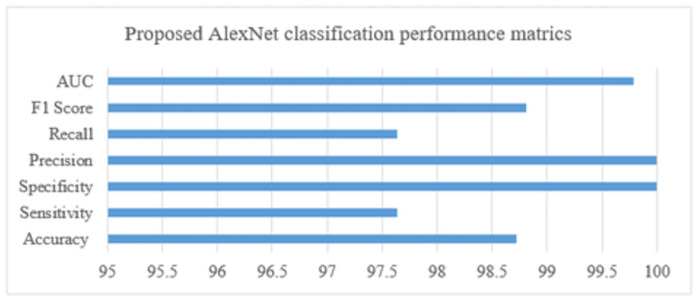
System performance metrics for tumor classification.

In Fig 13. The primary objective of this experiment is to assess the system framework’s use for multi-classification of pancreatic tumors (i.e., normal, pancreatic tumor, benign, pre-malignant, and malignant) using the AlexNet CNN model, which is based on transfer learning and produces good classification performance results. As can be seen in the graphical image below, the overall classification results were good, with 98.72 accuracy, 97.64 sensitivity, 100 specificity, 100 precision, 97.64 recall, 98.81 F1 score, and an area under the ROC curve (AUC) of 0.9979.

**Fig 14:**
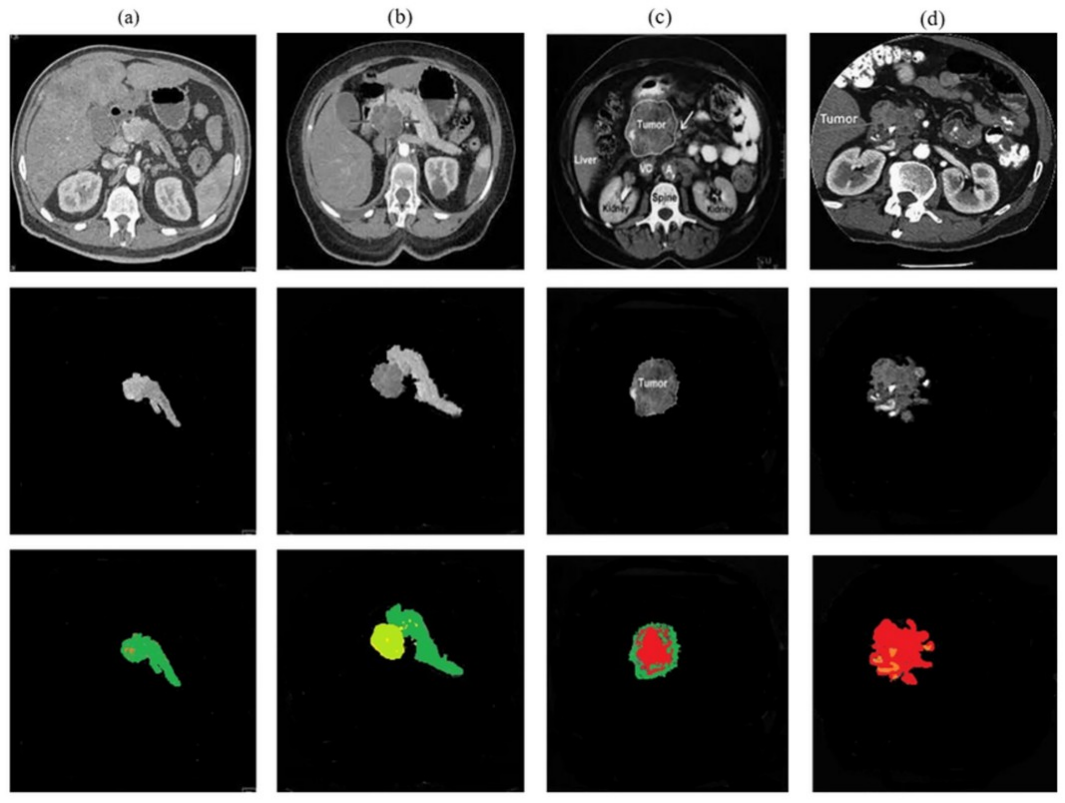
Pancreatic tumor classification results.

In the above Fig 14. The classification stages show how the detected tumor is effectively classified into normal, benign, pre-malignant, and malignant tumors. In (a) shows that the normal pancreas is classified with green color have no tumor. The benign tumor is elaborated in (b) and classified using the light green color and the green color shows the normal part of the pancreas. In (c) the pre-malignant tumor is classified and shows with red color that is going spread to the pancreatic cancerous tumor having an alarming condition. In (d) the malignant tumor is classified with a fully red color and shows the spread of cancerous cells of the tumor in the pancreas.

**Fig 15:**
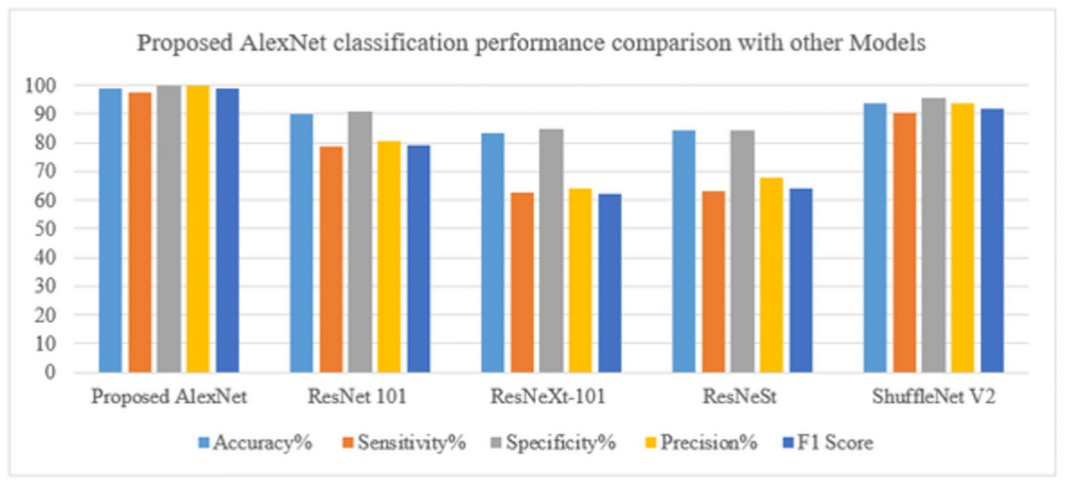
Comparison chart of performance metrics with other models for tumor classification.

**Fig 16:**
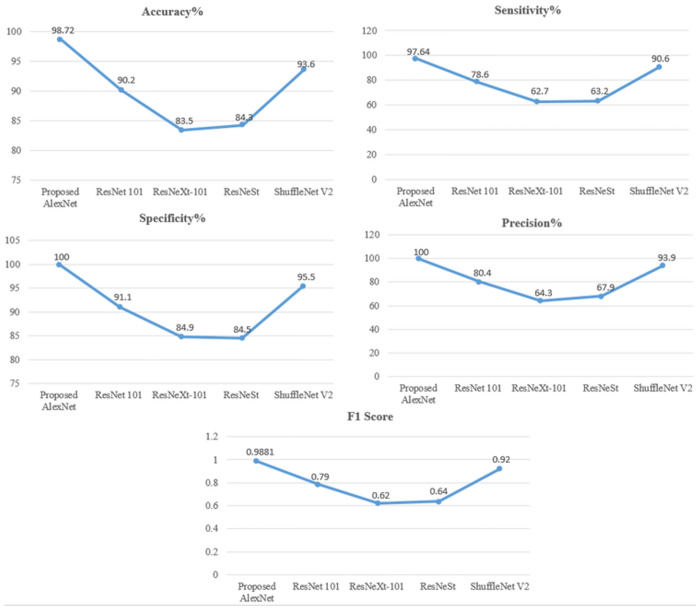
Comparison graph of performance evaluation with other models for tumor classification.

In Table 7. To show the superiority of our framework over current techniques, we created a multi-stage classification experiment system to compare our modal methodology with other existing pancreatic classification procedures. To do this, we made a comparison between our categorization framework and the most recent methods, the results of which are displayed in a table. Overall, the results of our suggested AlexNet classification model were good, with an F1 score of 0.9881, sensitivity of 97.64, specificity of 100, precision of 100, and accuracy of 98.72. [28] ResNet 101 has the following metrics: 80.4 precision, 91.1 specificity, 78.6 sensitivity, 90.2 accuracy, and 0.79 F1 score. The [29] findings for ResNeXt-101 show an F1 score of 0.62, accuracy of 83.5, sensitivity of 62.7, specificity of 84.9, and precision of 64.3. The [30] ResNeST model has an F1 score of 0.64, accuracy of 84.3, sensitivity of 63.2, specificity of 84.5, and precision of 67.9. As seen in Figs 15 and 16, and below, the ShuffleNet V2 has an accuracy of 93.6, sensitivity of 90.6, specificity of 95.5, precision of 93.9, and an F1 score of 0.92 in the most recent data.

**Table 7:** Classification performance metrics comparison of proposed and other models.

Model	Accuracy%	Sensitivity%	Specificity%	Precision%	F1 Score
Proposed AlexNet	98.72	97.64	100	100	0.9881
ResNet 101 [28]	90.2	78.6	91.1	80.4	0.79
ResNeXt-101 [29]	83.5	62.7	84.9	64.3	0.62
ResNeSt [30]	84.3	63.2	84.5	67.9	0.64
ShuffleNet V2	93.6	90.6	95.5	93.9	0.92

**Fig 17:**
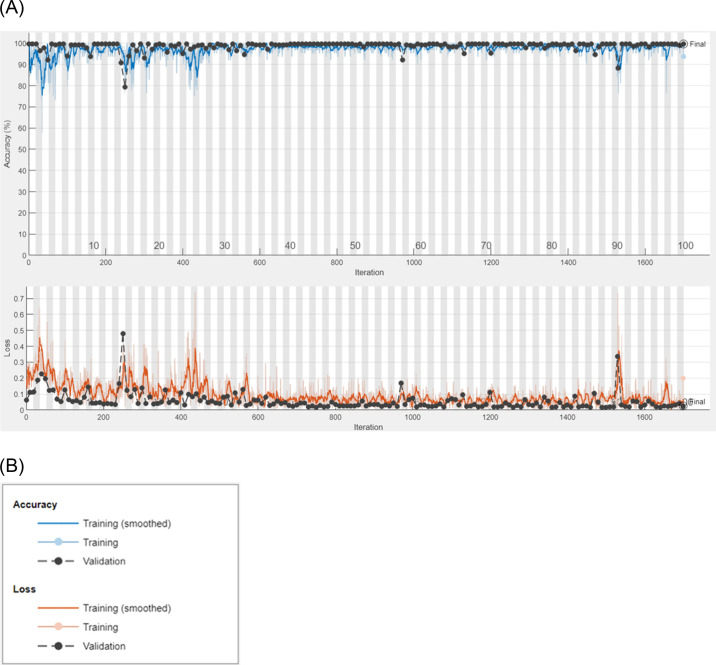
**A.** Training and validation Chart of accuracy and loss. **B.** Training and validation Chart of accuracy and loss.

Fig 17A and 17B in this chart show the accuracy and loss outcomes of the AlexNet CNN model’s whole training and validation process. Three distinct lines, the blue line representing the model’s smoothness, the light blue dotted line representing the model’s training, and the black dotted line representing the model’s validation illustrate the model’s correctness and efficiency in terms of performance. In the loss chart, the model’s smoothness is shown by the orange line, its training is indicated by the light orange dotted line, and its validation is indicated by the black dotted line.

**Fig 18:**
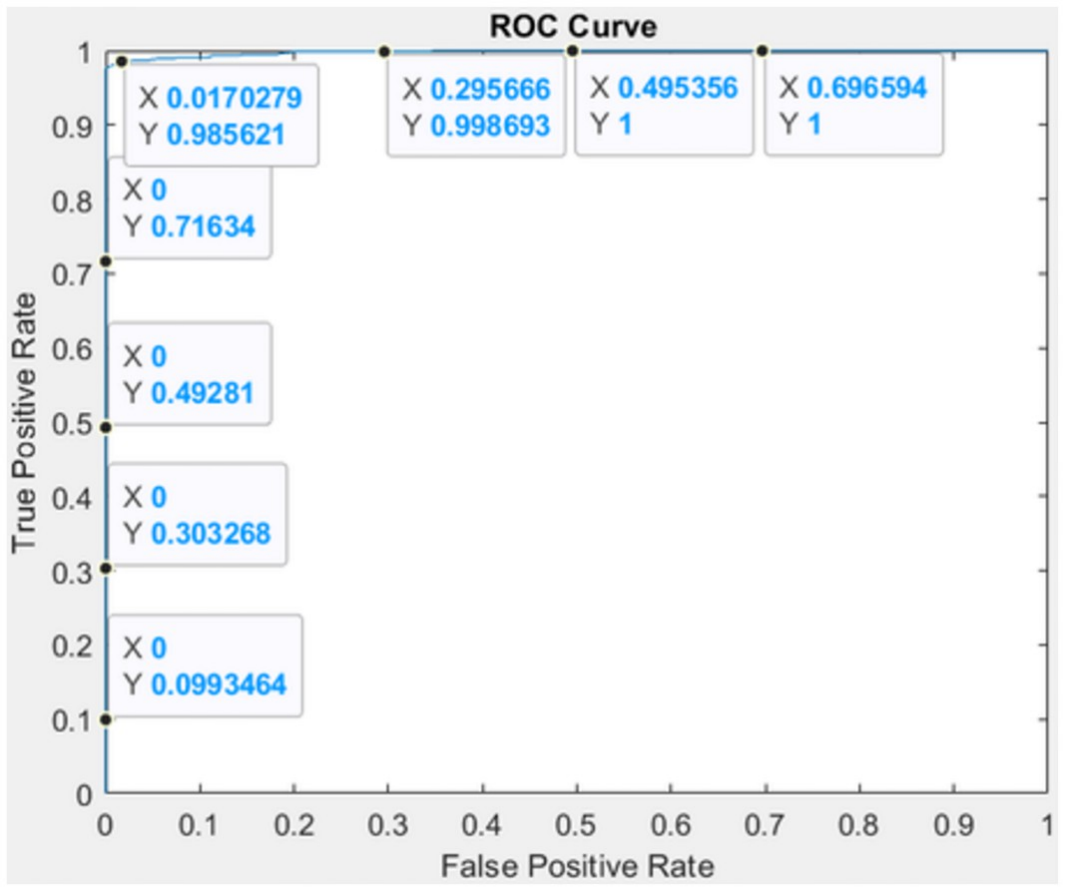
ROC curve for tumor classification.

In Fig 18. Certainly! Let’s delve deeper into the interpretation of ROC curve results. On the x-axis, the False Positive Rate (FPR) shows the percentage of negative cases. A low FPR indicates that the classifier is good at not labeling negative instances as positive. Interpretation of Points on the ROC Curve (0.0170279, 0.985621), (0.295666, 0.998693), (0.495356, 1), (0.696594, 1) As the FPR increases slightly, the TPR remains very high. This indicates that even when we allow for a small number of false positives, our classifier maintains a high TPR. This balance is crucial for a good classifier. The percentage of positive cases that are accurately categorized as positive is known as the True Positive Rate (TPR) (y-axis). The classifier’s ability to correctly identify positive cases as positive is demonstrated by a high TPR. The interpretation of the ROC Curve’s points (0, 0.0993464), (0, 0.303268), (0, 0.49281), and (0, 0.71634) indicates that our classifier obtain high TPRs at the beginning of the curve while maintaining a good FPR of 0. It means that even when we very strict about classifying something as positive (resulting in very few false positives), our classifier can still identify a significant portion of the true positives. Overall Performance of the ROC curve’s shape and the high TPR values across different FPRs indicate that our classifier performs well across various thresholds. It can maintain a high true positive rate while effectively controlling false positives. (AUC) The area under the Curve is a measure of how well our classifier can distinguish between classes. A higher AUC value (closer to 1) indicates better performance. Based on our ROC curve and the points provided, our classifier has a high AUC, which is a strong indicator of its overall performance. In summary, our ROC curve and the associated points suggest that our classifier performs very well, with low false-positive rates and high true-positive rates across different thresholds, leading to a high AUC.

The provided Table 8. Outlines the configuration and results of a neural network training process. (Sgdm) Stochastic Gradient Descent with Momentum is employed as the optimization algorithm, initializing with a learning rate of 0.001. The training data is split into mini-batches of 256 samples, with a maximum of 60 training epochs. Validation occurs every 100 iterations. The solver incorporates a momentum term set at 0.9, and there’s no explicit learning rate schedule, indicating a constant rate. L2 regularization with a coefficient of 0.0001 is applied for weight regularization. During training, input normalization is reset, and batch normalization statistics utilize population statistics. The training data is shuffled every epoch to enhance diversity. There is no early stopping, and the final model is based on the last iteration. Gradient clipping, using the L2 norm of gradients, has a threshold set to infinity. The training is set to an auto-execution environment, adapting to available hardware. Checkpoints are saved after each epoch, and the model achieved a final validation accuracy of 98.72%. The training, initiated on January 22, 2024, lasted 46 minutes and 40 seconds. The process concluded after 60 epochs and 240 iterations, reaching the predefined maximum. The model was trained on a single CPU, and the learning rate remained constant at 0.001 throughout the training period.

**Table 8:** AlexNet-CNN model training and validation details.

**Results**		**Training time**	
Validation accuracy	98.72%	Start time	22-Feb-2024, 22:07:51
Training finished	Max epoch complete	Elapsed time	2800 sec
**Training cycle**		**Solver**	
Epoch	60 of 60	Momentum	0.9
Iteration	240 of 240	**Other information**	
Iteration per epoch	4	Hardware resources	Single CPU
Maximum iteration	240	Learning rate schedule	Constant
**Learn Rate**		Learning rate	0.001
Learn Rate Schedule	None	**Frequently Used**	
Learn Rate Drop Factor	0.1	Solver	Sgdm
Learn Rate Drop Period	10	Initial Learn Rate	0.001
**Normalization and Regularization**		Mini Batch Size	256
L2Regularization	0.0001	Max Epochs	60
Reset Input Normalization	True	Validation Frequency	100
Batch Normalization Statistics	Population	**Gradient Clipping**	
**Mini-Batch**		Gradient Threshold Method	I2norm
Shuffle	Gradient Threshold	Gradient Threshold	Inf
**Checkpoint**		Hardware	
Checkpoint Path	Specify checkpoint path	Execution Environment	Auto
Checkpoint Frequency	1	**Validation and Output**	
Checkpoint Frequency Unit	epoch	Validation Patience	Inf
		Output Network	Last-iteration

#### Performance matrices

Therefore, we assess our system’s performance using these metrics about detection and classification. The ratio of correctly predicted instances (both positive and negative) to all instances in the dataset is known as accuracy. It gauges how accurate the model is overall. The variables that represent the number of true positives (TP), true negatives (TN), false positives (FP), and false negatives (FN) are the number of true positives (TP) (incorrectly predicted positive instances), and the number of false negatives (INR) (incorrectly predicted negative instances) as expressed in Eq (30).


$$Accuracy=\frac{T_{P}+T_{N}}{T_{P}+T_{N}+F_{P}+F_{N}}$$ (30)

This metric quantifies the percentage of real positive cases that the model accurately predicts. Another name for it is the true positive rate. as expressed in Eq (31).


$$Sensitivity=\frac{T_{P}}{T_{P}+F_{N}}$$ (31)

The percentage of real negative occurrences that the model accurately predicts is referred to as specificity. It is the true negative rate as expressed in Eq (32).


$$Specificity=\frac{T_{N}}{T_{N}+F_{P}}$$ (32)

Precision quantifies the percentage of favorable predictions that actually occur. It indicates the accuracy of positive predictions as expressed in Eq (33).


$$Precision=\frac{T_{P}}{T_{P}+F_{P}}$$ (33)

Recall is a metric that quantifies a classifier’s accuracy in identifying genuine positive instances among all actual positive cases in the dataset. It is sometimes referred to as the true positive rate or sensitivity. When positive examples must be detected and false negatives (missing positive instances) are more damaging than false positives (erroneously identifying negative instances as positive), recall becomes especially critical. For example, in medical diagnostics, it’s often more critical to correctly identify patients with a disease (true positives) even if it means some healthy patients are classified as having the disease (false positives). To summarize, recall assesses a classifier’s effectiveness in accurately identifying positive cases by measuring its capacity to recognize all relevant instances (true positives) among all real positive examples. In the context of a binary classification problem (e.g., detecting tumors as either benign or malignant), recall is calculated as expressed in Eq (34).


$$Recall=\frac{T_{P}}{T_{P}+F_{N}}$$ (34)

The harmonic mean of recall and accuracy is the F1 score. It offers a harmony between recall and accuracy as expressed in Eq (35).


$$F1\;Score=\frac{2*\left(precision*recall\right)}{precision+recall}$$ (35)

It is a single statistic that captures the essence of a classification model’s performance, particularly in cases when the distribution of classes is unbalanced. In conclusion, these metrics aid in assessing the effectiveness of a classification model by taking into account several factors, including total correctness (accuracy), sensitivity, specificity, and avoidance of false alarms; precision of positive predictions; and an equilibrium between precision and recall (F1 score).

## Discussion

The proposed CAD system for pancreatic tumor detection and classification demonstrated impressive performance metrics. The system achieved a detection accuracy of 99.64%, sensitivity of 99.17%, and specificity of 99.81%. These results indicate the robustness of the system in accurately identifying and localizing pancreatic tumors in CT images. Additionally, the classification stage attained an accuracy of 98.72% and an AUC of 0.9979, highlighting the system’s capability to effectively differentiate between normal, benign, pre-malignant, and malignant tumors. When compared to existing models, our system outperforms in several aspects. For instance, the VGG-CNN model reported an accuracy of 98.6%, sensitivity of 97.3%, and specificity of 100% with an AUC of 0.997. Although the VGG-CNN achieved a perfect specificity, our model shows a higher overall detection accuracy and comparable sensitivity. Similarly, other models like the Custom CNN Feature Pyramid and the Encoder-only CNN reported lower accuracies and AUC values, which highlights the superiority of our approach. The use of watershed segmentation combined with a reduced 11-layer AlexNet model contributed to the high performance metrics, providing a balance between computational efficiency and accuracy.

The proposed CAD system’s high accuracy and rapid processing time of 1.51 seconds per image make it a viable tool for clinical use. The preprocessing steps, including anisotropic diffusion filtering and morphological operations, ensured the preservation of image details while reducing noise, enhancing the accuracy of subsequent segmentation and classification stages. The ROC curve analysis further demonstrated the system’s reliability across various thresholds, with a high true positive rate and low false positive rate.

Our study shows how well deep learning can be used to identify and categorize pancreatic cancer in the Computer-aided Diagnosis (CAD) system. The system’s capacity to precisely detect and categorize pancreatic tumors is demonstrated by its excellent levels of accuracy, sensitivity, specificity, and area under the curve (AUC). By comparing our proposed methodology with other existing models, we show that our framework outperforms them in terms of detection and classification performance. This comparison highlights the superiority of our approach and its potential for clinical applications. The high performance of our CAD system has significant implications for the field of medical imaging and cancer diagnosis. It suggests that our system could be valuable for early detection, and monitoring of pancreatic cancer, potentially improving patient outcomes. The detailed experimental setups and system specifications provided in our research ensure transparency and reproducibility, which are essential for scientific research. This allows other researchers to validate and build upon our findings.

In summary, the proposed CAD system shows significant potential in assisting radiologists by providing accurate and efficient detection and classification of pancreatic tumors. The comparative analysis with existing models underscores the system’s advancements and sets a benchmark for future research in automated tumor detection and classification using deep learning techniques.

## Conclusion

Our research concludes that the proposed CAD (computer-aided diagnosis) system for pancreatic cancer detection and classification using deep learning is effective, achieving good performance results. The proposed system aims to address the limitations of the manual identification of pancreatic tumors by radiologists, which is challenging and time-consuming due to the complex nature of CT scan images. The objective of the work is to apply a deep learning model to create a four-stage framework for the preprocessing, segmentation, detection, and classification of pancreatic cancers. The potential for this discovery to transform early pancreatic cancer detection and classification makes it significant. The suggested CAD system can greatly increase diagnostic efficiency and accuracy by automating the tumor identification and categorization process. This might result in early detection and the potential to save many lives worldwide. The study contributes to the advancement of medical imaging and cancer diagnosis by offering a promising approach for early detection and classification of pancreatic cancer. In summary, our framework integrates state-of-the-art image processing and deep learning techniques to automate the detection and classification of tumors in CT images, providing a valuable tool for improving diagnostic accuracy and efficiency in clinical settings.

## Limitations and future work

Despite the promising results, there are some limitations to our study. First, the dataset used for training and validation was relatively small, which may limit the generalizability of the model. Future work should focus on expanding the dataset to include a more diverse set of images from different sources and populations to ensure the model’s robustness across various clinical settings. Second, the system currently processes CT images; however, integrating multimodal imaging data, such as MRI and PET scans, could further enhance the accuracy and reliability of tumor detection and classification. Future research should explore the fusion of different imaging modalities to create a more comprehensive diagnostic tool.

Another limitation is the computational resources required for training the deep learning model. Although the proposed system is efficient in processing time, the initial training phase is resource-intensive. Optimizing the training process and exploring more lightweight models could make the system more accessible for clinical implementation. Lastly, while the system achieved high accuracy in classification, the differentiation between pre-malignant and malignant tumors could be further improved. Incorporating additional clinical features, such as patient history and genetic information, may provide a more holistic approach to tumor classification and improve diagnostic accuracy.

In future work, we aim to address these limitations by expanding the dataset, integrating multimodal imaging, optimizing the model architecture, and incorporating additional clinical data. This will not only enhance the system’s performance but also ensure its practical applicability in diverse clinical environments.

Subsequent research endeavors investigate sophisticated deep learning architectures, including transformer-based models or attention processes, to improve the CAD system’s detection and classification capabilities. Integrating multi-modal imaging data, such as combining CT with MRI or PET scans, could enhance the effectiveness and robustness of the CAD system. Further validation on larger and more diverse datasets, including data from different demographics and geographical regions, could enhance the generalizability of the proposed methodology. The findings’ applicability to different populations or imaging procedures be limited by their dependence on a single dataset for training and testing. Overall, our research highlights the potential of the suggested CAD system for enhancing clinical practice in the fields of medical imaging and cancer diagnosis by demonstrating its efficacy for pancreatic cancer detection and categorization.

## References

[1] Sung H. et al., "Global cancer statistics 2020: GLOBOCAN estimates of incidence and mortality worldwide for 36 cancers in 185 countries," *CA*: *a cancer journal for clinicians*, vol. 71, no. 3, pp. 209–249, 2021. doi: 10.3322/caac.21660 33538338

[2] Howlader N. et al., "SEER cancer statistics review, 1975–2018," *National Cancer Institute*, pp. 1–25, 2021.

[3] J. Ferlay, I. Soerjomataram, and M. Ervik, "Dikshit, r," *Eser*, *S*., *Mathers*, *c*., *rebelo*, *M*., *Parkin*, *DM*, *Forman*, *D*., *Bray*, *F*. *GLOBO-can2012 v1*. *0*, *cancer Incidence and Mortality Worldwide*: *Iarc cancerBase*, no. 11, 2012. 10.1002/ijc.29210 25220842

[4] Hidalgo M. et al., "Addressing the challenges of pancreatic cancer: future directions for improving outcomes," *Pancreatology*, vol. 15, no. 1, pp. 8–18, 2015. doi: 10.1016/j.pan.2014.10.001 25547205

[5] Vincent A., Herman J., Schulick R., Hruban R. H., and Goggins M., "Pancreatic cancer," *The lancet*, vol. 378, no. 9791, pp. 607–620, 2011. 10.1016/S0140-6736(10)62307-0 PMC3062508 21620466

[6] Dinesh M., Bacanin N., Askar S., and Abouhawwash M., "Diagnostic ability of deep learning in detection of pancreatic tumour," *Scientific Reports*, vol. 13, no. 1, p. 9725, 2023. doi: 10.1038/s41598-023-36886-8 37322046 PMC10272117

[7] N. Bhanja, A. Akila, D. Sudheer, A. Kumar, P. Chanda, and R. Dani, "Modified Cuckoo Algorithm (mCA-CNN) for Detection and Diagnosis of Pancreatic Tumor using Region-based Segmentation Techniques," *in 2023 2nd International Conference on Applied Artificial Intelligence and Computing (ICAAIC)*, 2023: IEEE, pp. 1003–1008.

[8] Chen P.-T. et al., "Pancreatic cancer detection on CT scans with deep learning: a nationwide population-based study," *Radiology*, vol. 306, no. 1, pp. 172–182, 2023. doi: 10.1148/radiol.220152 36098642

[9] Huang L. et al., "Resection of pancreatic cancer in Europe and USA: an international large-scale study highlighting large variations," *Gut*, vol. 68, no. 1, pp. 130–139, 2019. doi: 10.1136/gutjnl-2017-314828 29158237

[10] van Leeuwen K. G., de Rooij M., Schalekamp S., van Ginneken B., and Rutten M. J., "How does artificial intelligence in radiology improve efficiency and health outcomes?," *Pediatric Radiology*, pp. 1–7, 2021. 34117522 10.1007/s00247-021-05114-8 PMC9537124

[11] Malamateniou C., Knapp K., Pergola M., Woznitza N., and Hardy M., "Artificial intelligence in radiography: where are we now and what does the future hold?," *Radiography*, vol. 27, pp. S58–S62, 2021. doi: 10.1016/j.radi.2021.07.015 34380589

[12] Montagnon E. et al., "Deep learning workflow in radiology: a primer," *Insights into imaging*, vol. 11, pp. 1–15, 2020. 32040647 10.1186/s13244-019-0832-5 PMC7010882

[13] Hosny A., Parmar C., Quackenbush J., Schwartz L. H., and Aerts H. J., "Artificial intelligence in radiology," *Nature Reviews Cancer*, vol. 18, no. 8, pp. 500–510, 2018. doi: 10.1038/s41568-018-0016-5 29777175 PMC6268174

[14] Liu X. et al., "A comparison of deep learning performance against health-care professionals in detecting diseases from medical imaging: a systematic review and meta-analysis," *The lancet digital health*, vol. 1, no. 6, pp. e271–e297, 2019. doi: 10.1016/S2589-7500(19)30123-2 33323251

[15] de Groof A. J. et al., "Deep-learning system detects neoplasia in patients with Barrett’s esophagus with higher accuracy than endoscopists in a multistep training and validation study with benchmarking," *Gastroenterology*, vol. 158, no. 4, pp. 915–929. e4, 2020. doi: 10.1053/j.gastro.2019.11.030 31759929

[16] Takhar A. S., Palaniappan P., Dhingsa R., and Lobo D. N., "Recent developments in diagnosis of pancreatic cancer," *Bmj*, vol. 329, no. 7467, pp. 668–673, 2004. doi: 10.1136/bmj.329.7467.668 15374918 PMC517650

[17] Zavalsız M. T., Alhajj S., Sailunaz K., Özyer T., and Alhajj R., "A comparative study of different pre-trained deeplearning models and custom CNN for pancreatic tumor detection," *International Arab Journal of Information Technology*, 2023.

[18] McKinney S. M. et al., "International evaluation of an AI system for breast cancer screening," *Nature*, vol. 577, no. 7788, pp. 89–94, 2020. doi: 10.1038/s41586-019-1799-6 31894144

[19] Esteva A. et al., "Dermatologist-level classification of skin cancer with deep neural networks," *nature*, vol. 542, no. 7639, pp. 115–118, 2017. doi: 10.1038/nature21056 28117445 PMC8382232

[20] Yasaka K., Akai H., Abe O., and Kiryu S., "Deep learning with convolutional neural network for differentiation of liver masses at dynamic contrast-enhanced CT: a preliminary study," *Radiology*, vol. 286, no. 3, pp. 887–896, 2018. doi: 10.1148/radiol.2017170706 29059036

[21] Krizhevsky A., Sutskever I., and Hinton G. E., "Imagenet classification with deep convolutional neural networks," *Advances in neural information processing systems*, vol. 25, 2012.

[22] Ramaekers M. et al., "Computer-Aided Detection for Pancreatic Cancer Diagnosis: Radiological Challenges and Future Directions," *Journal of Clinical Medicine*, vol. 12, no. 13, p. 4209, 2023. doi: 10.3390/jcm12134209 37445243 PMC10342462

[23] Liu K.-L. et al., "Deep learning to distinguish pancreatic cancer tissue from non-cancerous pancreatic tissue: a retrospective study with cross-racial external validation," *The Lancet Digital Health*, vol. 2, no. 6, pp. e303–e313, 2020. doi: 10.1016/S2589-7500(20)30078-9 33328124

[24] Z. Zhang, S. Li, Z. Wang, and Y. Lu, "A novel and efficient tumor detection framework for pancreatic cancer via CT images," in *2020 42nd Annual International Conference of the IEEE Engineering in Medicine & Biology Society (EMBC)*, 2020: IEEE, pp. 1160–1164. 10.1109/EMBC44109.2020.9176172 33018193

[25] Ma H. et al., "Construction of a convolutional neural network classifier developed by computed tomography images for pancreatic cancer diagnosis," *World Journal of Gastroenterology*, vol. 26, no. 34, p. 5156, 2020. doi: 10.3748/wjg.v26.i34.5156 32982116 PMC7495037

[26] Si K. et al., "Fully end-to-end deep-learning-based diagnosis of pancreatic tumors," *Theranostics*, vol. 11, no. 4, p. 1982, 2021. doi: 10.7150/thno.52508 33408793 PMC7778580

[27] Z. Zhu, Y. Xia, L. Xie, E. K. Fishman, and A. L. Yuille, "Multi-scale coarse-to-fine segmentation for screening pancreatic ductal adenocarcinoma," in *Medical Image Computing and Computer Assisted Intervention—MICCAI 2019*: *22nd International Conference*, *Shenzhen*, *China*, *October 13–17*, *2019*, *Proceedings*, *Part VI 22*, 2019: Springer, pp. 3–12.

[28] K. He, X. Zhang, S. Ren, and J. Sun, "Deep residual learning for image recognition," in *Proceedings of the IEEE conference on computer vision and pattern recognition*, 2016, pp. 770–778.

[29] S. Xie, R. Girshick, P. Dollár, Z. Tu, and K. He, "Aggregated residual transformations for deep neural networks 2017 IEEE Conference on Computer Vision and Pattern Recognition (CVPR)," *IEEE*, 2017.

[30] H. Zhang et al., "Resnest: Split-attention networks," in *Proceedings of the IEEE/CVF conference on computer vision and pattern recognition*, 2022, pp. 2736–2746.

[31] Manabe K., Asami Y., Yamada T., and Sugimori H., "Improvement in the convolutional neural network for computed tomography images," *Applied Sciences*, vol. 11, no. 4, p. 1505, 2021.

[32] Liu S.-L. et al., "Establishment and application of an artificial intelligence diagnosis system for pancreatic cancer with a faster region-based convolutional neural network," *Chinese medical journal*, vol. 132, no. 23, pp. 2795–2803, 2019. doi: 10.1097/CM9.0000000000000544 31856050 PMC6940082

[33] Sekaran K., Chandana P., Krishna N. M., and Kadry S., "Deep learning convolutional neural network (CNN) With Gaussian mixture model for predicting pancreatic cancer," *Multimedia Tools and Applications*, vol. 79, no. 15–16, pp. 10233–10247, 2020.

[34] Huang B. et al., "Artificial intelligence in pancreatic cancer," *Theranostics*, vol. 12, no. 16, p. 6931, 2022. doi: 10.7150/thno.77949 36276650 PMC9576619

[35] Ghorpade H. et al., "Automatic Segmentation of Pancreas and Pancreatic Tumor: A Review of a Decade of Research," *IEEE Access*, 2023.

[36] Yang M. et al., "AX-Unet: A deep learning framework for image segmentation to assist pancreatic tumor diagnosis," *Frontiers in Oncology*, vol. 12, p. 894970, 2022. doi: 10.3389/fonc.2022.894970 35719964 PMC9202000

[37] Zhang C., Achuthan A., and Himel G. M. S., "State-of-the-Art and Challenges in Pancreatic CT Segmentation: A Systematic Review of U-Net and Its Variants," *IEEE Access*, 2024.

[38] Gandikota H. P., "CT scan pancreatic cancer segmentation and classification using deep learning and the tunicate swarm algorithm," *Plos one*, vol. 18, no. 11, p. e0292785, 2023. doi: 10.1371/journal.pone.0292785 37930963 PMC10627451

[39] Zhang Z., Tian H., Xu Z., Bian Y., and Wu J., "Application of a pyramid pooling Unet model with integrated attention mechanism and Inception module in pancreatic tumor segmentation," *Journal of Applied Clinical Medical Physics*, vol. 24, no. 12, p. e14204, 2023. doi: 10.1002/acm2.14204 37937804 PMC10691628

[40] Alves N., Schuurmans M., Litjens G., Bosma J. S., Hermans J., and Huisman H., "Fully automatic deep learning framework for pancreatic ductal adenocarcinoma detection on computed tomography," *Cancers*, vol. 14, no. 2, p. 376, 2022. doi: 10.3390/cancers14020376 35053538 PMC8774174

[41] Patel H., Zanos T., and Hewitt D. B., "Deep Learning Applications in Pancreatic Cancer," *Cancers*, vol. 16, no. 2, p. 436, 2024. doi: 10.3390/cancers16020436 38275877 PMC10814475

[42] Viriyasaranon T. et al., "Annotation-Efficient Deep Learning Model for Pancreatic Cancer Diagnosis and Classification Using CT Images: A Retrospective Diagnostic Study," *Cancers*, vol. 15, no. 13, p. 3392, 2023. doi: 10.3390/cancers15133392 37444502 PMC10340780

[43] Vaiyapuri T. et al., "Intelligent deep-learning-enabled decision-making medical system for pancreatic tumor classification on CT images," in *Healthcare*, 2022, vol. 10, no. 4: MDPI, p. 677. doi: 10.3390/healthcare10040677 35455854 PMC9027672

